# Chaperoning of specific tau structure by immunophilin FKBP12 regulates the neuronal resilience to extracellular stress

**DOI:** 10.1126/sciadv.add9789

**Published:** 2023-02-01

**Authors:** Lulu Jiang, Pijush Chakraborty, Lushuang Zhang, Melissa Wong, Shannon E. Hill, Chelsea Joy Webber, Jenna Libera, Laura J. Blair, Benjamin Wolozin, Markus Zweckstetter

**Affiliations:** ^1^ Department of Pharmacology and Experimental Therapeutics, Boston University School of Medicine, Boston, MA 02118, USA.; ^2^ German Center for Neurodegenerative Diseases (DZNE), Von-Siebold-Str. 3a, 37075 Göttingen, Germany.; ^3^ Department of Molecular Medicine, College of Medicine, Byrd Alzheimer’s Institute, University of South Florida, Tampa, FL 33612, USA.; ^4^ Center for Neurophotonics, Boston University, Boston, MA 02215, USA.; ^5^ Center for Systems Neuroscience, Boston University, Boston, MA 02215, USA.; ^6^ Department for NMR-based Structural Biology, Max Planck Institute for Multidisciplinary Sciences, Am Faßberg 11, 37077 Göttingen, Germany.

## Abstract

Alzheimer’s disease and related tauopathies are characterized by the pathogenic misfolding and aggregation of the microtubule-associated protein tau. Understanding how endogenous chaperones modulate tau misfolding could guide future therapies. Here, we show that the immunophilin FKBP12, the 12-kDa FK506-binding protein (also known as FKBP prolyl isomerase 1A), regulates the neuronal resilience by chaperoning a specific structure in monomeric tau. Using a combination of mouse and cell experiments, in vitro aggregation experiments, nuclear magnetic resonance–based structural analysis of monomeric tau, site-specific phosphorylation and mutation, as well as structure-based analysis using the neural network–based structure prediction program AlphaFold, we define the molecular factors that govern the binding of FKBP12 to tau and its influence on tau-induced neurotoxicity. We further demonstrate that tyrosine phosphorylation of tau blocks the binding of FKBP12 to two highly specific structural motifs in tau. Our data together with previous results demonstrating FKBP12/tau colocalization in neurons and neurofibrillary tangles support a critical role of FKBP12 in regulating tau pathology.

## Teaser

Molecular studies shed light on the mechanisms behind the enigmatic role of immunophilin FKBP12 in AD and other tauopathies.

## INTRODUCTION

The microtubule-associated protein tau (MAPT) is the major constituent of the intracellular neurofibrillary tangles that are a pathological hallmark of Alzheimer’s disease (AD) and other associated tauopathies. In healthy conditions, tau stabilizes the microtubule assembly. However, under pathological conditions, or in response to stress, tau becomes hyperphosphorylated and forms oligomers, leading to the degeneration of the structure and function of the nervous system (*1*, *2*). Tau pathology correlates strongly with neurodegeneration and cognitive decline in AD (*3*).

Tau is intrinsically disordered, and different proteins that constrain the structural freedom of tau are reported to be essential for its processing and engage in its accumulation (*4*–*7*). Among the many interacting partners of tau, peptidyl-prolyl cis-trans isomerases (PPIases) play an important role in regulating tau activity and stability thereby influencing its aggregation into amyloid fibrils (*8*–*10*). PPIases, which are a unique family of molecular chaperones, were originally identified for the ability to bind the compound FK506 and mediate immunosuppression, with FKBP12 (the 12-kDa FK506-binding protein) being the only member of this family that is essential for the immunosuppressant actions of FK506 (*11*, *12*). Four PPIases have been implicated in tau biology. FKBP51 and 52 regulate the turnover of native tau protein, which affects the levels of tau in neurons (*4*, *13*). FKBP12 and Pin1 are PPIases that directly regulate tau misfolding by regulating proline isomerization in the central microtubule-interacting region. Pin1 binds to tau in a phosphorylation-dependent manner (*14*). In contrast, FKBP12 does not require tau phosphorylation for binding and prevention of tau aggregation (*15*–*18*). The ability of FKBP12 to recognize tau before it gets hyperphosphorylated and rapidly oligomerizes makes FKBP12 a critical regulator in preventing tau aggregation (*15*–*18*).

FKBP12, a 12-kDa immunophilin, is a cytosolic protein with a high degree of expression in neuronal cell bodies and neurites (*18*). FKBP12 colocalizes with tau in neurons and neurofibrillary tangles (*18*). In addition, FKBP12 binds to the Aβ oligomers and regulates the processing of amyloid precursor protein (*19*). In the brain of patients with AD, the expression of FKBP12 decreases with an altered localization to neuropil threads, reactive astrocytes, and dystrophic neurites (*18*). Recent genetics and proteomes studies with AD brains also indicated the decrease of FKBP12 in their dataset (*20*, *21*). The cellular reduction and redistribution of FKBP12 might be responsible for the aberrant phosphorylation of tau, leading to the formation of neurofibrillary tangles. The mechanism through which FKBP12 regulates tauopathy is, however, poorly understood. The small number of studies investigating the interaction of FKBP12 with tau has documented that it can prevent tau aggregation in vitro, but little is known about the mechanisms through which this protection occurs (*15*–*18*).

In this study, we provide high-resolution insight into the interaction between tau and FKBP12. We investigate the expression of FKBP12 in a mouse model of tauopathy expressing P301S tau. We demonstrate colocalization with tau pathology and that overexpressing FKBP12 prevents the formation of tau pathology in primary cortical neurons. We then dissect the molecular mechanism of FKBP12 action on tau through the combination of in vitro aggregation assays, nuclear magnetic resonance (NMR)–based identification of a specific structural motif in monomeric tau, site-specific phosphorylation and mutation, as well as structure-based analysis using the neural network–based structure prediction software AlphaFold (*22*). At last, with a three-dimensional (3D) human neuron-astrocyte assembloid tauopathy model, we demonstrated that overexpression of FKBP12 can strongly reduce tau pathology, which leads to a concomitant decrease in neurodegeneration.

## RESULTS

### FKBP12 progressively decreases in P301S Tau PS19 mice brain

**Fig. 1.**
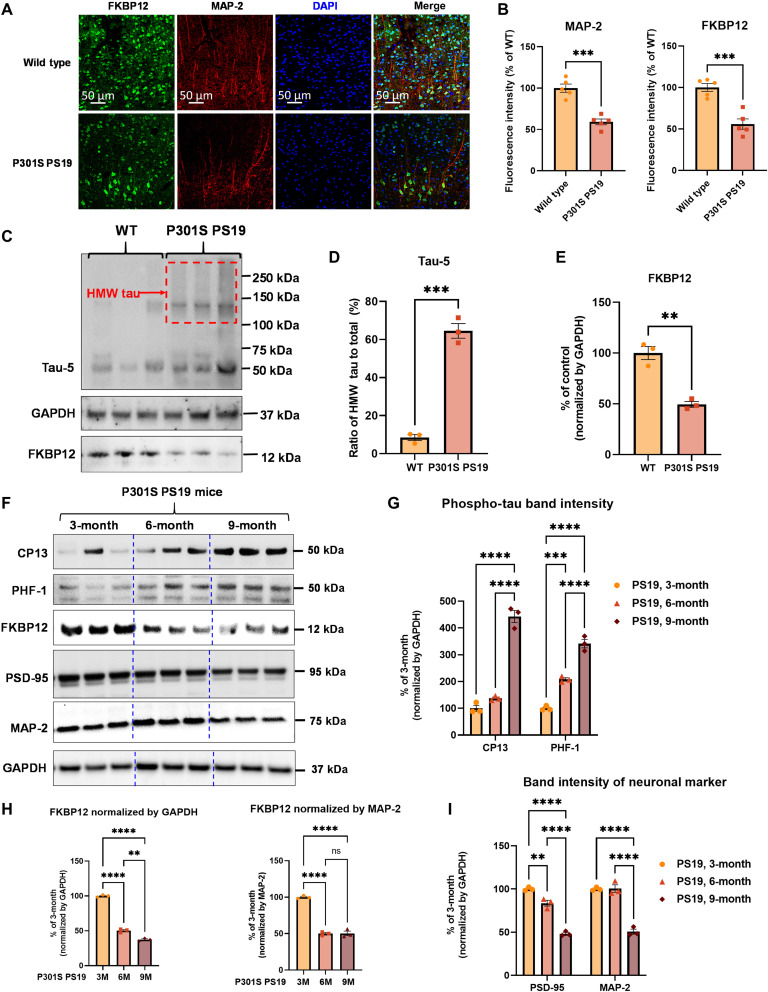
FKBP12 is decreased in PS19 mice brain. (**A**) Representative images showing the distribution of FKBP12 in entorhinal cortex of mouse brain. Scale bars, 50 μm. (**B**) Quantification of MAP-2 and FKBP12 fluorescence intensity in entorhinal cortex of PS19 mice brain in comparison to age-matched wild type (WT). Data are expressed as means ± SEM. *N* = 5. Statistics by unpaired *t* test, ***P < 0.005. (**C**) Representative Western blot showing high–molecular weight (HMW) tau aggregation in PS19 mice brain lysate and the decrease of FKBP12 in comparison to wild type. GAPDH, glyceraldehyde-3-phosphate dehydrogenase. (**D**) Quantification of the tau-5 band intensity showing the robust increase of the ratio between high–molecular weight tau to monomeric tau. (**E**) Quantification of the FKBP12 band intensity. (**F**) Representative Western blot images showing the expression level of phosphorylated tau (CP13 and PHF-1), FKBP12, and neuronal markers [postsynaptic density 95 (PSD-95) and MAP-2] in the brain lysate of 3-, 6-, and 9-month-old PS19 mice, respectively. (**G**) Quantification of the phosphorylated tau (CP13 and PHF-1) band intensity. Result was normalized by internal control of corresponding GAPDH band intensity. (**H**) Quantification of FKBP12 Western blot (WB) band intensity normalized by GAPDH and MAP-2, respectively. Statistics by one-way analysis of variance (ANOVA), post hoc multiple comparisons test by Tukey’s test. (**I**) Quantification for the band intensity of neuronal markers including PSD-95 and MAP-2, normalized by GAPDH. Data are expressed as means ± SEM. *N* = 3. Statistics by two-way ANOVA, and post hoc multiple comparisons test by Tukey’s. **P < 0.01, ***P < 0.005, and ****P < 0.001.

To explore the role of FKBP12 in tauopathy neurons, we explored the biology of FKBP12 in a mouse model overexpressing human P301S Tau (PS19 mice). Colabeling of FKBP12 (green) with the neuronal marker MAP-2 (red) showed strong reduction of FKBP12 in aged 9-month-old PS19 mice, which is a point at which neurons are lost in the entorhinal cortex (Fig. 1, A and B). We also examined the aggregation state of tau in the 9-month-old PS19 mice using native polyacrylamide gel electrophoresis (PAGE) gels. Our result showed that the brains of 9-month-old PS19 mice developed high–molecular weight tau aggregates and a robust decrease of FKBP12 compared to age-matched control (Fig. 1, C to E). To further analyze whether the reduced FKBP12 is corelated to tau pathology or the result of neuronal loss, we examined the FKBP12 levels in younger PS19 mice. Total brain lysates were collected from 3-, 6-, and 9-month-old PS19 mice. The expression of phosphorylated tau, by CP13 (Tau phos Ser202) and PHF-1 (Tau phos Ser396/Ser404) antibody, respectively, FKBP12 and neuronal markers (postsynaptic density 95/PSD-95 and MAP-2) were measured by Western blot. Our result showed that the decrease of FKBP12 correlates with the progression of tau pathology and precedes neuronal loss (Fig. 1, F to I).

### FKBP12 translocates from axonal hillock to soma and colocalizes to tau inclusions after seeding of oligomeric tau

To further investigate the interaction between tau aggregates and FKBP12, we imaged the distribution of FKBP12 in neurons exhibiting oligomeric tau (oTau) accumulation. Primary cortical neurons were plated in 24-well plate and transduced with human 4R0N tau with Adeno-Associated Virus Serotype 1 (AAV1) on day 4. After 14 days in vitro, 40 ng of oTau (S1p fraction extracted from aged PS19 mice) was added into the culture medium to induce tau seeding as described previously (*23*, *24*).

**Fig. 2.**
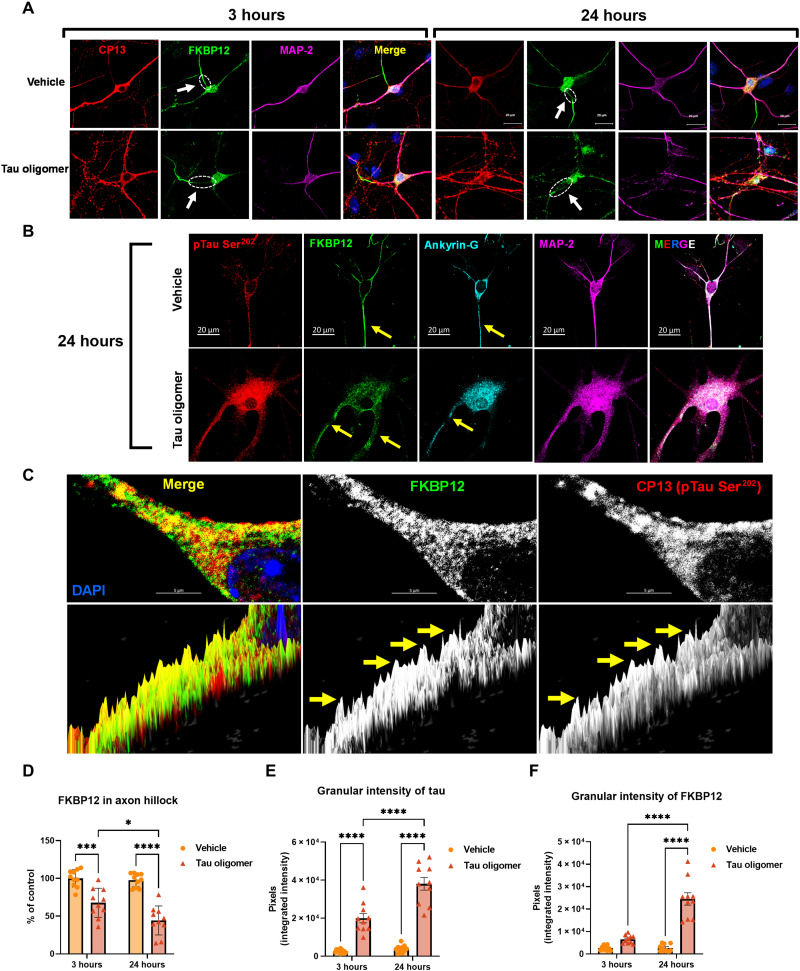
FKBP12 was translocated from axonal hillock to soma and colocalized to oTau. (**A**) Representative images of tau phosphorylation (CP13 antibody, red) and FKBP12 translocation in primary cortical neurons after induction of tau aggregation by oligomeric S1p fraction. Scale bars, 20 μm. (**B**) Representative images showed the high expression level of FKBP12 (green) in axonal hillock/axon initial segment (labeled by anti–ankyrin-G antibody, bright blue) under basal conditions whereas FKBP12 translocated to soma and dendrites when neurons bear tau aggregation. Scale bars, 20 μm. (**C**) Representative images showed the spatial colocalization of FKBP12 and aggregated Tau in the neurons after 24 hours of oTau seeding. Scale bars, 5 μm. DAPI, 4′,6-diamidino-2-phenylindole. (**D**) Quantification of FKBP12 intensity in the axon hillock of the neurons at 3 and 24 hours of S1p treatment, respectively. Data are expressed as means ± SEM. *N* = 10. Statistics by two-way ANOVA, post hoc multiple comparisons test by Fisher’s least significant difference (LSD). *P < 0.05, ***P < 0.005, and ****P < 0.001. (**E** and **F**) Quantification of granular intensity of CP13-labeled tau aggregates (E) and FKBP12 (F) in neurons at 3 and 24 hours of S1p treatment, respectively. Data are expressed as means ± SEM. *N* = 10. Statistics by two-way ANOVA, and post hoc multiple comparisons test by Fisher’s LSD. ****P < 0.001.

After exposure of the neurons to oTau for 3 or 24 hours, the neurons were fixed and examined for the tau pathology and FKBP12, respectively. Our result showed that, under normal conditions, FKBP12 is highly concentrated in the axonal hillocks of neurons [Fig. 2, A and B, arrow points to axonal hillock, labeled by anti–ankyrin-G antibody (*25*)]. However, short-term (3-hour) exposure to oTau elicited translocation of FKBP12 from axonal hillock to the soma of the affected neurons (Fig. 2A). By 24 hours after exposure to oTau, the FKBP12 was redistributed to soma and dendrites and colocalized with phosphorylated tau (Fig. 2, B and C, arrow points to axonal hillock, labeled by anti–ankyrin-G antibody). Higher-magnification images further showed that the phospho-tau labeling is granular, suggesting that the tau was aggregated and that the FKBP12 had been recruited into these tau aggregates (Fig. 2C). Quantification of FKBP12 intensity showed that FKBP12 was selectively reduced in the axonal hillock but increased in the granular tau region (Fig. 2, D to F).

### FKBP12 potentiates the resilience of neurons to stress response

**Fig. 3.**
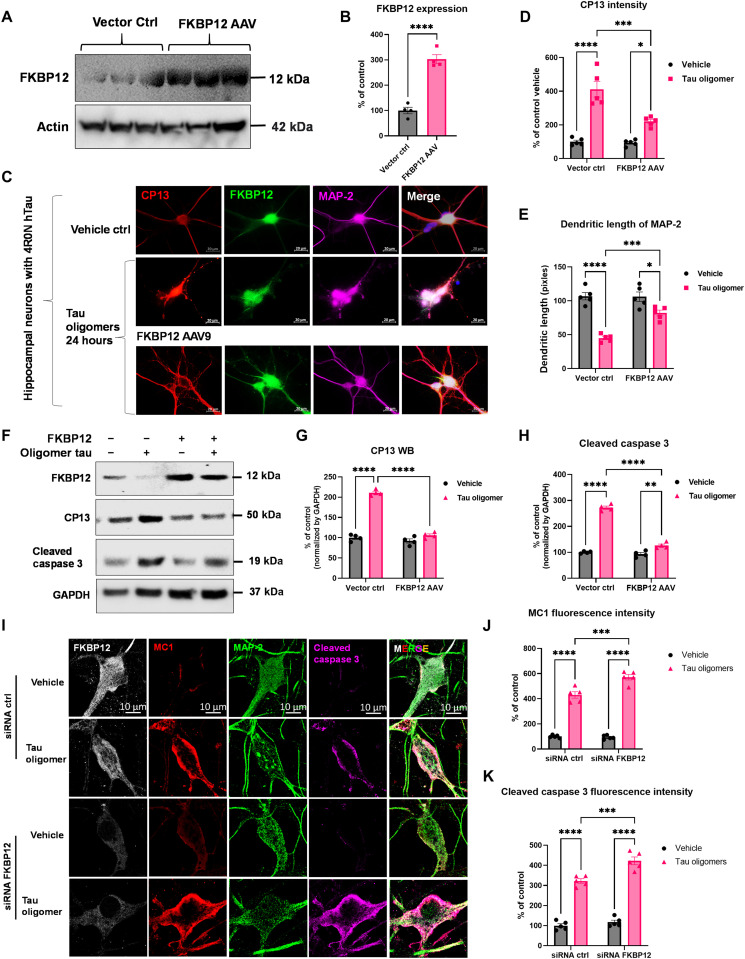
FKBP12 potentiates the resilience of neurons to stress response. (**A**) Representative blot image showed the elevation of FKBP12 in neurons transduced with FKBP12 AAV9. (**B**) Quantification of FKBP12 in neuronal lysate overexpressed with AAV9 vector. Data are expressed as means ± SEM. *N* = 3. Statistics by unpaired t test, ****P < 0.001. (**C**) Representative fluorescence-labeled images showed that overexpression of FKBP12 (green) preserved the integrity of neurons (labeled by MAP-2, magenta) and reduced the granular intensity of tau (labeled by CP13, red). Neurons were fixed at 24 hours after oTau stimulation. Scale bars, 20 μm. (**D**) Quantification of CP13 granular intensity as shown in (C). *P < 0.05, ***P < 0.005, and ****P < 0.001. ctrl, control. (**E**) Quantification of neuronal dendritic length labeled by MAP-2 as shown in (C). Data were collected from five independent experiments, expressed as means ± SEM. *P < 0.05, ***P < 0.005, ****P < 0.001. (**F**) Representative blot image showed the expression level of FKBP12, phosphorylated tau detected by CP13 (Tau phos Ser^202^) and cleaved caspase 3 in neurons transduced with FKBP12 AAV9 and/or stressed by tau oligomers. GAPDH is examined as the internal control. (**G** and **H**) Quantification of CP13 and cleaved caspase 3 band intensity. Data were normalized by GAPDH, shown as % of basal condition without FKBP12 overexpression and oTau stress, and expressed as means ± SEM. *N* = 3. **P < 0.01and ****P < 0.001. (**I**) Representative fluorescence colabeling images showed that FKBP12 knock down by small interfering RNA (siRNA) accelerated and potentiated the neuronal apoptosis (by cleaved caspase 3, magenta) induced by tau oligomers. (**J** and **K**) Quantification of MC1 (red) and cleaved caspase 3 fluorescence intensity as shown in (F), respectively. Data were collected from five independent experiments, expressed as means ± SEM. Statistics by two-way ANOVA, and post hoc multiple comparisons test by Fisher’s LSD. ***P < 0.005 and ****P < 0.001.

We hypothesized that FKBP12 stabilizes the association of tau with microtubules and lack of FKBP12 potentiates tau misfolding and aggregation. To investigate the role of FKBP12 in tau aggregation, we overexpressed or knocked down FKBP12 in the primary neurons. Primary cortical neurons from C57 mice were transduced with human 4R0N tau (AAV1) at day in vitro 3 (DIV-3) and FKBP12 (AAV9) at DIV-5, respectively. At DIV-14, the neurons were treated with oTau (S1p fractions of PS19 brain lysate) for 24 hours followed by fixation or snap freezing. AAV-mediated transduction increased FKBP12 expression threefold in cortical cell culture (Fig. 3, A and B). Overexpression of FKBP12 reduced the accumulation of phosphorylated tau, as detected with the anti–phospho-tau antibody CP13 (tau phospho-Ser^202^) (Fig. 3, C to E). In addition, we observed a corresponding preservation of the integrity of neurons labeled by MAP-2 (Fig. 3, C to E). To further validate the effect of FKBP12 overexpression on tau pathology and neuronal damage, we also collected the cell lysate from the neurons with AAV transduction and oTau challenge, followed by Western blot analysis. The biochemical characterization of tau pathology (labeled by CP13 antibody, pTau Ser^202^) and neuronal apoptosis (by cleaved caspase 3 antibody) showed that overexpression of FKBP12 was capable of inhibiting tau phosphorylation and reducing accumulation of cleaved caspase 3 (Fig. 3, F to H).

To test the role of endogenous FKBP12 in tau aggregation, we examined the effects of small interfering RNA–mediated FKBP12 knockdown in DIV-6 cortical neurons. Imaging showed that FKBP12 normally colocalizes with misfolded tau (labeled by MC1, a conformation dependent antibody, epitope within amino acids 312 to 322) in neurons exposed to oTau (Fig. 3, I to K). Neurons exposed to oTau also exhibited increased apoptotic markers as shown by cleaved caspase 3 labeling in oTau-treated neurons (Fig. 3, I and K). Reducing FKBP12 led to elevated levels of tau pathology, as shown by MC1 levels, and accumulation of cleaved caspase 3, indicative of cell death (Fig. 3, I to K). These results indicate that FKBP12 can protect against tau aggregation and that the lack of FKBP12 enhances the neurotoxicity induced by tau oligomerization.

### FKBP12 binds a unique structural motif in monomeric tau

**Fig. 4.**
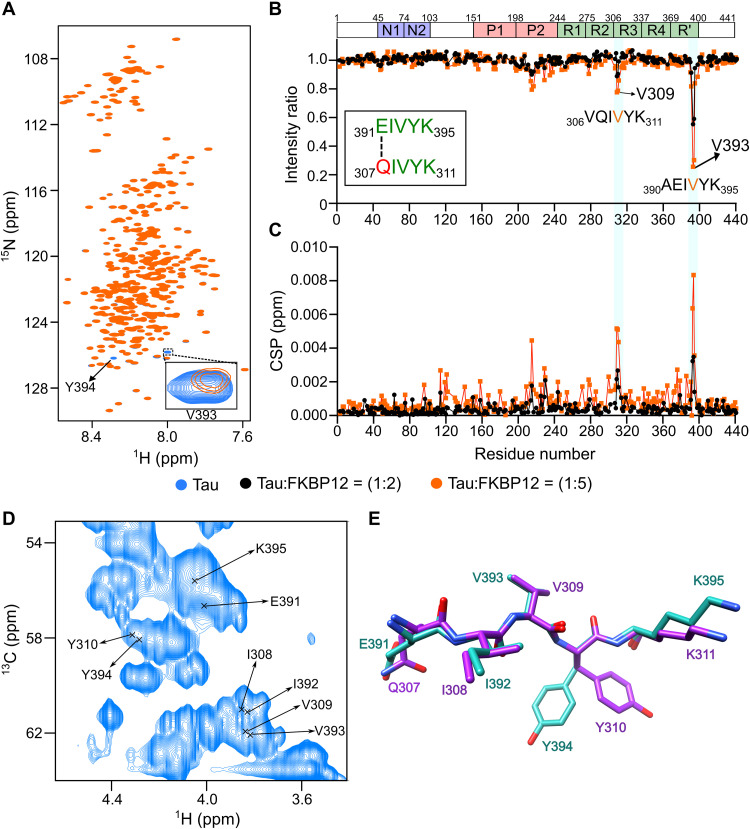
FKBP12 binds a unique structural motif in monomeric tau. (**A**) 2D ^1^H-^15^N HSQC spectra of tau in the absence (blue) or fivefold excess (orange) of FKBP12. The FKBP12-induced broadening of V393 is highlighted in the inset. ppm, parts per million. (**B** and **C**) Changes in the intensities (B) and chemical shift perturbations (CSPs) (C) of cross peaks in the HSQC spectra of tau upon addition of twofold (black) and fivefold (orange) excess of FKBP12. The sequence similarity of the two interaction sites of tau is displayed in the inset in (B). The domain organization of tau is shown on above. (**D**) Selected region of the 2D ^1^H-^13^C HSQC spectrum of tau. The Cα-Hα assignments of ^391^EIVYK^395^ and ^308^IVY^310^ are marked. Assignments of Q307 and K311 are not available. (**E**) Superposition of the preferred solution conformations of the two tau sequences ^307^QIVYK^311^ (purple) and ^391^EIVYK^395^ (cyan) to which FKBP12 binds.

To determine the molecular basis of the interaction of tau with FKBP12, we recorded 2D NMR correlation spectra. Two- and fivefold molar excess of FKBP12 was added to NMR-observable 4R tau. Tau’s ^1^H-^15^N NMR spectrum was highly similar without and with FKBP12 (Fig. 4A). This shows that tau is largely disordered when interacting with FKBP12 (*26*). However, we observed a selective change in the intensity and position of tau residues from ^307^QIVYK^311^ and ^391^EIVYK^395^ (Fig. 4, A to C). In addition, residues ^215^LPTPP^219^, in the proline-rich region P2, weakly sensed FKBP12 (Fig. 4, B and C). Because the strength of NMR signal perturbation is linked to the binding affinity, it shows that FKBP12 preferentially binds to the two short sequences ^307^QIVYK^311^ and ^391^EIVYK^395^ in the 441-residue tau protein. The two sequences do not contain proline, differ in a single residue, and form the motif xIVYK. No similar sequence motif is present in other parts of tau.

The highly selective nature of the tau/FKBP12 interaction might root in a specific structure in monomeric tau. Consistent with this hypothesis, we find highly similar signal patterns for the two five-residue sequences in 2D ^1^H-^13^C spectra (Fig. 4D). To determine this structure, we estimated the backbone dihedral angles from the experimental N, Co, and Ca chemical shifts. The dihedral angles define two extended structures in ^307^QIVYK^311^ and ^391^EIVYK^395^ (Fig. 4E). Superposition of the two structures reveals a common structural motif (Fig. 4E) with altering side-chain orientations of I308/I392, V309/V393, and Y310/Y394 (Fig. 4E). The binding of monomeric tau to FKBP12 thus engages a specific structural motif.

### Structure of the tau/FKBP12 complex

**Fig. 5.**
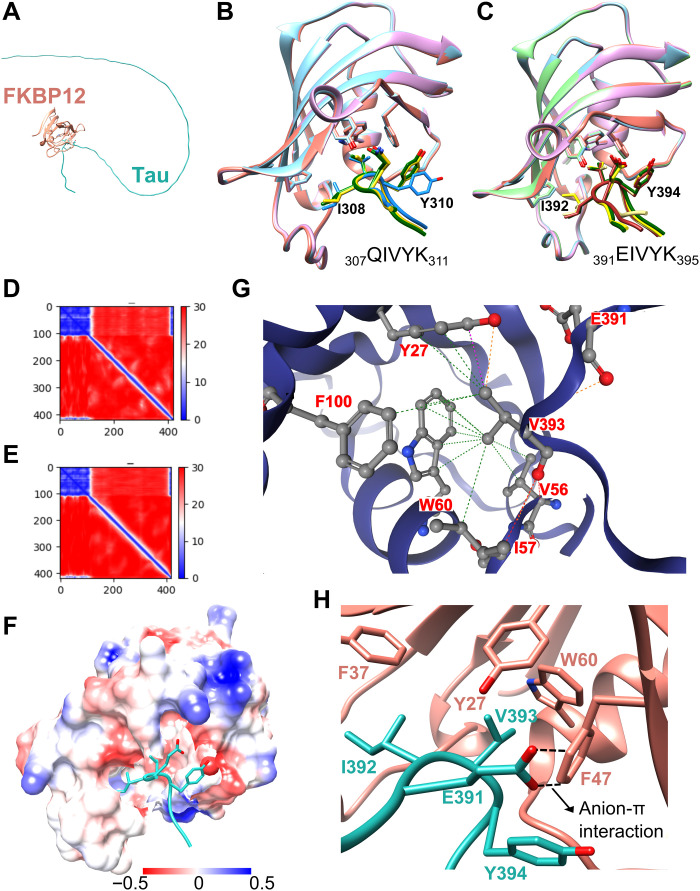
Structural model of the tau/FKBP12 complex. (**A**) Schematic representation of FKBP12 (orange) interacting with the disordered tau protein (cyan). (**B** and **C**) Ensemble of complex structures predicted by AlphaFold2 for the interaction of FKBP12 with ^307^QIVYK^311^ (B) and ^391^EIVYK^395^ (C) of tau. The three (B) and four (C) most similar peptide conformations (from five calculated models) are shown. (**D** and **E**) Residue-specific predicted alignment error (*22*) plots generated by AlphaFold2 for one of the representative complex structures shown in (B) and (C), respectively. Because FKBP12 and the tau sequences were connected by a 300-residue poly-glycine linker for the AlphaFold2 calculations, FKBP12 residues are found at positions 1 to 108 and the tau sequences at positions 409 to 419. (**F** to **H**) Structure of ^391^EIVYK^395^ of tau (cyan) bound to the hydrophobic surface of FKBP12 as predicted by AlphaFold2 (*22*). The electrostatic surface potential of FKBP12 is displayed in (F) with positive and negative charges shown in blue and red, respectively. The interaction interface is highlighted in (G) and (H). The hydrophobic interaction of V393 of tau with F100, W60, V56, and Y27 of FKBP12 is shown in (G). The anion-π interaction between E391 of tau and F47 of FKBP12 is marked by dashed lines in (H).

To gain high-resolution insight into the tau/FKBP12 complex, we made use of the recent breakthrough in structure prediction (*22*). The neural network software AlphaFold2 predicts highly accurate structures of protein/peptide complexes when sufficient evolutionary constraints/prior information is available (*27*, *28*). The availability of specific binding site information is particularly critical when modeling complexes with large disordered proteins where most of the protein remains disordered in the bound state. This is also the case for the tau/FKBP12 complex (Fig. 5A). However, we identified two highly specific FKBP12-binding sites in tau (^307^QIVYK^311^ and ^391^EIVYK^395^). We can further simplify the structural analysis by taking into account their nearly identical amino acid sequences: Because of the high sequence similarity, it is likely that only one of the two sequences will be bound to FKBP12 at any time.

We modeled the complex structures of FKBP12 with tau’s ^307^QIVYK^311^ (Fig. 5B) and ^391^EIVYK^395^ (Fig. 5C). The structure of FKBP12, as seen in complex with the peptides, deviates by 0.4 Å from the experimental structure of FKBP12 [Protein Data Bank ID 1FKJ (*29*)]. In addition, the residue-specific predicted alignment errors were low for both FKBP12 and the tau peptides (Fig. 5, D and E), suggesting a high accuracy of the modeled complex structures.

Tau’s ^307^QIVYK^311^ and ^391^EIVYK^395^ bind to the hydrophobic pocket of FKBP12 (Fig. 5, B, C, and F). The hydrophobic pocket is conserved in the family of FKBP proteins and forms the catalytic site of peptidyl-prolyl cis-trans isomerization (*30*). The binding of ^307^QIVYK^311^ to FKBP12’s catalytic center is supported by previous mutational analysis. FKBP12 carrying a mutation in the catalytic pocket is unable to block aggregation of a 31-residue oligopeptide containing ^307^QIVYK^311^ (*15*). Despite lacking proline, the two specific structural motifs in tau thus bind to the active site of FKBP12.

### Interactions stabilizing the tau/FKBP12 complex

In the structure of the tau(^391^EIVYK^395^)/FKBP12 complex, the side chain of V393 deeply inserts into FKBP12’s hydrophobic pocket (Fig. 5G). The pocket is formed by Y27, V56, W60, and F100. The complex is further stabilized by an anion-π interaction between E391 of tau and F47 of FKBP12 (Fig. 5H). In anion-π interactions, the positively charged edge of an aromatic ring interacts with the anion of the glutamate side chain, forming a favorable anion-quadrupole interaction (*31*).

The structure of the tau(^307^QIVYK^311^)/FKBP12 complex displays similar stabilizing interactions with the V309 side chain inside FKBP12’s hydrophobic pocket (Fig. 5B). However, at position i-2 with respective to the central valine (V309 or V393) a glutamine is present. This glutamine replaces the glutamic acid of ^391^EIVYK^395^, which forms the anion-π interaction with F47 of FKBP12 (Fig. 5H). The lack of this interaction is likely to disfavor complex formation of FKBP12 with ^307^QIVYK^311^ when compared to ^391^EIVYK^395^. Support for this is provided when we computationally analyze the E391Q-associated stability change using DynaMut2 (*32*). The mutation is predicted to destabilize the complex structure by −0.18 kcal/mol. The computational analysis is further in agreement with weaker FKBP12-induced NMR signal perturbation for residues ^307^QIVYK^311^ when compared to ^391^EIVYK^395^ in tau (Fig. 4, B and C).

### Tau tyrosine phosphorylation blocks FKBP12 binding

**Fig. 6.**
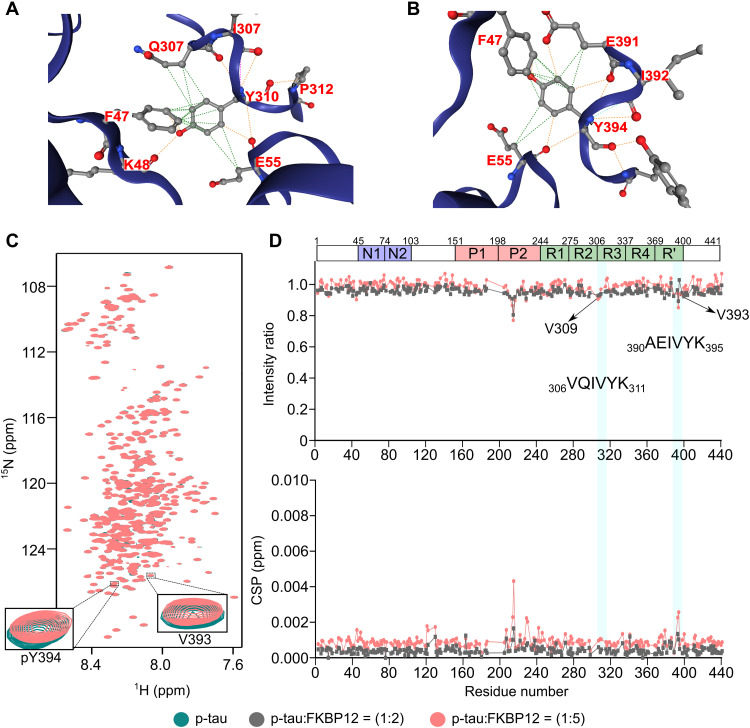
Tyrosine phosphorylation blocks tau interaction with FKBP12. (**A** and **B**) Contacts between Y310 (A) or Y394 (B) of tau with F47 and E55 of FKBP12. (**C**) 2D ^1^H-^15^N HSQC spectra of cAbl-phosphorylated tau in the absence (green) or fivefold excess (brick red) of FKBP12. The NMR signals of V393 and pY394 are highlighted in the inset. (**D**) Changes in the intensities (top) and CSPs (bottom) of cross peaks in the HSQC spectra of cAbl-phosphorylated tau upon addition of twofold (gray) or fivefold (brick red) excess of FKBP12. The domain organization of tau is shown on top.

Tau aggregates in the brain are hyperphosphorylated (*33*). Phosphorylation occurs at serine, threonine, and tyrosine (*33*). The tyrosine kinase cAbl efficiently phosphorylates Y18, Y197, Y310, and Y394 (*34*, *35*). Y310 and Y394 are in the two structural motifs that are recognized by FKBP12 (Fig. 4). In the modeled complex structures with FKBP12, the tyrosines’ aromatic rings make contacts with FKBP12’s F47 and E55 (Fig. 6, A and B). In addition, an intramolecular contact with either E391 or Q307 is stabilizing the complex (Fig. 6, A and B). The importance of the tyrosine residue in the FKBP12-binding motif of tau is further supported by the DynaMut2-predicted stability decrease of −2.11 kcal/mol upon mutation of Y310 to asparagine. This replacement reflects the difference between the amino acid sequence of ^307^QIVYK^311^, the FKBP12-binding motif in pseudo-repeat R3, and the homologous sequence ^276^QIINK^280^ in pseudo-repeat R2. NMR spectroscopy indicated that ^276^QIINK^280^ binds much weaker to FKBP12 (Fig. 4, B and C), supporting the computational analysis and thus the importance of the tyrosine residue in the xIVYK motif for binding to FKBP12.

To gain insight into the impact of tau tyrosine phosphorylation on the binding to FKBP12, we phosphorylated 4R tau with cAbl in vitro. Previous NMR-based quantification showed that cAbl phosphorylates 4R tau at the four tyrosine residues Y18, Y197, Y310, and Y394 (*35*). NMR spectra of the cAbl-phosphorylated 4R tau alone and with two- and fivefold molar FKBP12 excess were recorded (Fig. 6C). The NMR signals of the residues ^215^LPTPP^219^ in the proline-rich region P2 displayed similar FKBP12-induced chemical shift and intensity changes (Fig. 6, C and D) as in the unmodified tau protein (Fig. 4, B and C). This is expected, because no tyrosine residue is present in this region. In contrast, the NMR signals of V393 and the phosphorylated Y394 did not show any change in intensity and only a slight shift in signal position (Fig. 6, C and D). Moreover, the NMR signals of residues ^307^QIVYK^311^ did not respond to the addition of FKBP12 (Fig. 6, C and D). The analysis shows that phosphorylation of Y310 and Y394 inhibits the binding of FKBP12 to the two specific structural motifs in monomeric tau.

To further characterize the role of the tyrosine residues (Y310 and Y394) present in the two structural motifs of monomeric tau in FKBP12 binding, we mutated Y394 to asparagine. A tyrosine-to-asparagine mutation was selected because the homolog region in repeat R2 of tau contains an asparagine. Upon titrating the Y394N-mutant tau with two- and fivefold molar FKBP12 excess, we observed a similar pattern of chemical shift and intensity changes (fig. S1, A to C) for the residues ^215^LPTPP^219^ and ^307^QIVYK^311^ as in the unmodified tau protein (Fig. 4, B and C). However, the NMR signals of the residues ^391^EIVNK^395^ did not respond to the addition of the FKBP12 (fig. S1, B and C). This shows that the amyloid-like motif ^307^QIVYK^311^ of monomeric tau can still interact with FKBP12 in the absence of binding to the ^391^EIVYK^395^ motif.

### FKBP12 modulates tau aggregation

**Fig. 7.**
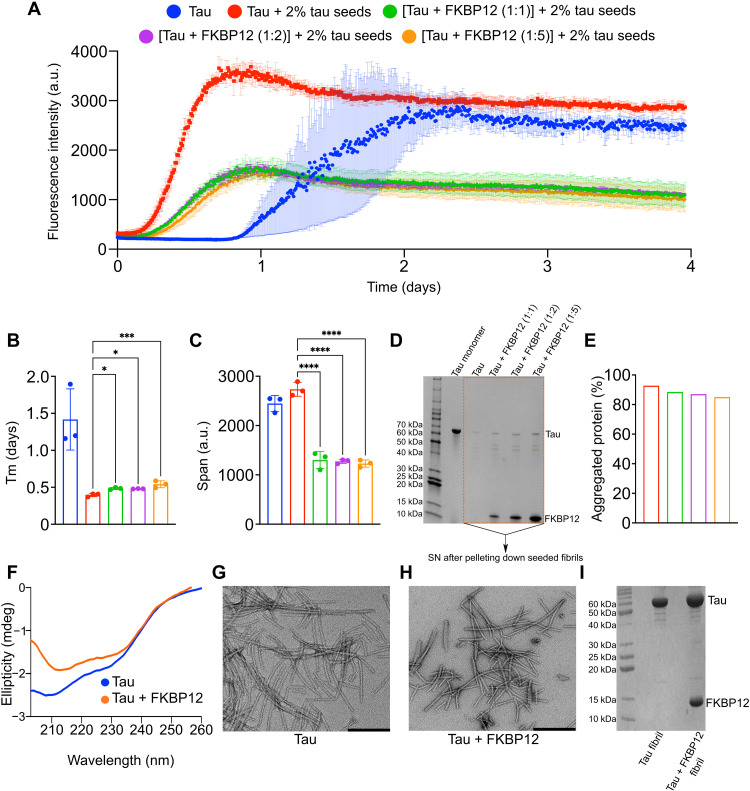
FKBP12 modulates tau aggregation in vitro. (**A**) Aggregation kinetics of 25 μM tau either in the absence (blue) or presence (red) of 2% preformed tau seeds. Aggregation kinetics of 1:1 (green), 1:2 (purple), and 1:5 (yellow) mixture of tau:FKBP12 in the presence of 2% preformed tau seeds. *N* = 3 per condition. a.u., arbitrary units. (**B**) Half time of aggregation (Tm) of de novo aggregated tau (blue) or seeded tau in the absence (red) or presence of equimolar (green), twofold (purple), or fivefold (yellow) molar excess of FKBP12. *P ([tau + 2% tau seeds] versus {[tau + FKBP12 (1:1)] + 2% tau seeds}) = 0.0104, *P ([tau + 2% tau seeds] versus {[tau + FKBP12 (1:2)] + 2% tau seeds}) = 0.0137, ***P = 0.0005. (**C**) Span of ThT intensity in the aggregation curves of de novo aggregated tau (blue) or seeded tau in the absence (red) or presence of equimolar (green), twofold (purple), or fivefold (yellow) molar excess of FKBP12. ****P < 0.0001. (**D**) SDS-PAGE gel of the monomeric tau protein and the supernatant (SN) of the seeded tau fibrils (after centrifugation) either in the absence or presence of equimolar, twofold, or fivefold excess of FKBP12. The fibril samples were taken after the 4 days of aggregation, as shown in (A). (**E**) The amount of protein aggregated. The % of aggregated proteins was determined by dividing the intensity of the SN to the monomeric tau. (**F**) CD spectra of tau fibrils formed without (blue) and with fivefold FKBP12 excess (orange). (**G** and **H**) Negative-stain EM image of tau fibril (G) + fivefold FKBP12 excess (H). Scale bars, 200 nm. (**I**) SDS-PAGE gel of tau fibrils without and with fivefold FKBP12 excess. Data are represented as means ± SD of *n* = 3 samples. Statistical analyses were performed by one-way ANOVA.

To better understand the impact of the direct interaction between tau and FKBP12 on tau aggregation, we performed in vitro fibrillization assays of tau using a tau seeding assay (*36*, *37*). The transneuronal propagation of tau pathology from an affected region of the brain to a healthy region occurs through “templated misfolding” in which the pathological tau seeds lead to the aggregation of monomeric tau protein (*36*). To investigate the role of FKBP12 on seeded tau aggregation, we added 2% preformed tau fibrils to monomeric tau protein in the absence or presence of FKBP12 (Fig. 7A). In the absence of FKBP12, the addition of tau seeds decreased the half time of aggregation (Fig. 7, A and B), confirming the seeding activity of tau fibrils. In addition, after 4 days of incubation, both the de novo aggregated and the seeded tau fibrils reached comparable thioflavin T (ThT) values (Fig. 7C). However, the presence of either equimolar, twofold, or fivefold excess of FKBP12 delayed the aggregation (Fig. 7, A and B) compared to the seeded aggregation of tau in the absence of FKBP12. In addition, all the samples with FKBP12 reached very similar ThT intensity at saturation, which was only half that of tau without FKBP12 (Fig. 7, A and C).

We confirmed the changes in seeded tau fibrillization using a centrifugation assay (Fig. 7D). The amount of tau protein aggregated in the presence or absence of FKBP12 was determined by centrifuging the fibril samples at the end of the incubation period and loading the supernatants (SNs) into an SDS-PAGE gel to analyze the residual soluble protein. Analysis of tau protein levels upon incubation with FKBP12 showed more soluble tau in the SN (Fig. 7, D and E).

Next, we examined a de novo tau fibrillization assay using a heparin-free fibrillization assay (*37*). These results also demonstrated that FKBP12 reduced tau aggregation, although the data needed to be examined using multiple different approaches to arrive at this conclusion. 4R tau started to form amyloid fibrils after ~15 hours (fig. S2A) (*37*). In the presence of an equimolar concentration or twofold molar FKBP12 excess, the time until robust ThT intensity was detected, i.e., when pronounced fibrillization occurred, was unchanged (fig. S2A). However, the fibrils grew slightly more rapidly in the presence of FKBP12 with lower ThT intensity at saturation (fig. S2, C and D). This suggests that an equimolar concentration or twofold molar excess of FKBP12 promotes the elongation of tau fibrils. We then increased the concentration of FKBP12 to fivefold molar excess. We monitored the aggregation kinetics (fig. S2A) and detected an increase in the fibrillization lag phase (fig. S2B). Large excess of FKBP12 thus delays tau fibrillization, although the ThT intensity reached at saturation was approximately doubled (fig. S2C). Further experiments using ThT-free approaches suggested that the higher steady-state tau fibril-ThT intensity might reflect increased association of tau fibrils with ThT rather than the accumulation of a larger amount of total tau fibrils (*37*).

Similar to the studies in Fig. 7D, we used centrifugation to explore whether the changes in steady-state ThT reflected changes in the interaction of tau with the ThT or changes in the total amount of fibrillar tau. The amount of tau protein aggregated in the presence or absence of FKBP12 was determined by centrifuging the fibril samples at the end of the incubation period and loaded the SNs into an SDS-PAGE gel to analyze the residual soluble protein. Analysis of tau protein levels upon incubation with FKBP12 showed more soluble tau in the SN (fig. S2E) and less aggregated protein in the pellet (fig. S2F). Notably, the amount of tau protein aggregated in the presence of one-, two-, and fivefold molar excess of FKBP12 was comparable (fig. S2, E and F). This suggests that the higher ThT intensity detected in the presence of fivefold molar excess of FKBP12 in fig. S2A is not due to the presence of more aggregates but due to the formation of aggregates with different morphology. We suggest that the presence of FKBP12 on the surface of tau fibrils either leads to the formation of fibrils with potentially different morphology or attenuates the binding of ThT.

To investigate morphological differences between de novo tau fibrils formed in the absence and presence of FKBP12, we recorded circular dichroism (CD) spectra and negative-stain electron microscopy (EM) images. CD demonstrated amyloid-like β-structure (Fig. 7F). The CD spectrum of the tau/FKBP12 mixture depends on both the tau fibril structure and the structure of FKBP12. Negative-stain EM revealed long fibrils composed of both twisted and straight tau filaments in the absence of FKBP12 (Fig. 7G). The tau fibrils formed in the presence of fivefold molar excess of FKBP12 also displayed twisted and straight morphology (Fig. 7H). However, they were shorter when compared to FKBP12-free tau fibrils (Fig. 7H). An inhibitory effect of FKBP12 on tau fibril growth is further supported by the smaller elongation rate of tau fibrils in the presence of FKBP12 (fig. S2, A and D). Thus, multiple independent lines of evidence indicate that FKBP12 acts to inhibit tau fibril assembly.

FKBP12 accumulates in tau tangles in the brain of patients with AD (*18*). The concentrations of FKBP12 are up to 3 μM in brain tissue, which is more than three times of endogenous tau level (*38*). This is consistent with the ratio range that we are testing in the current experiment. To test whether FKBP12 directly binds to tau fibrils, we pelleted the tau fibrils formed in the absence and presence of FKBP12 (fig. S2A). SDS-PAGE confirmed the presence of insoluble tau (Fig. 7I). In addition, a strong FKBP12 band was detected (Fig. 7I). FKBP12 thus binds to tau fibrils without additional binding partners. The binding of FKBP12 to tau fibrils can be rationalized on the basis of the interaction mechanism of FKBP12 with monomeric tau (Figs. 4 and 5). The identified FKBP12-binding motif ^391^EIVYK^395^ is located outside of the cross–β-structure core of all known tau fibril structures (*39*–*44*). It is thus likely to be accessible in tau aggregates. In addition, binding of FKBP12 to the ^215^LPTPP^219^ residues in the proline-rich domain P2 of tau might contribute to the association of FKBP12 with tau fibrils.

### Overexpression of FKBP12 inhibits tau pathology and prevents neurodegeneration in a 3D human neuron-astrocyte assembloid tauopathy model

**Fig. 8.**
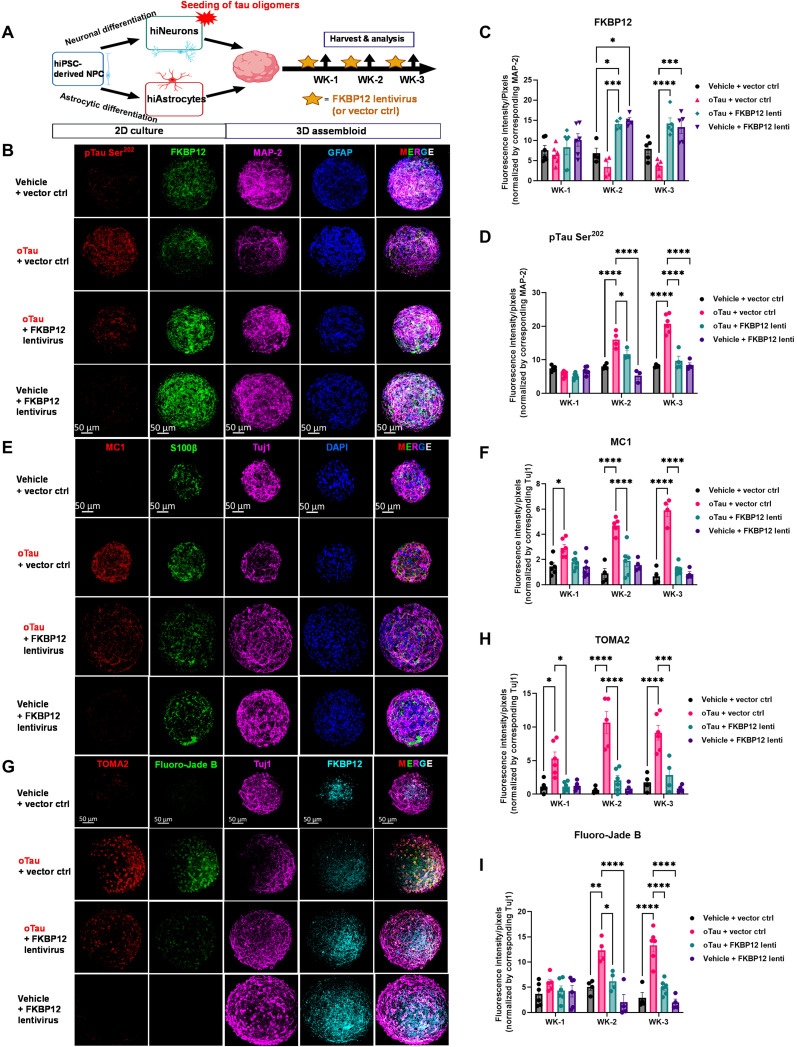
Overexpression of FKBP12 inhibits tau pathology and prevents neurodegeneration. (**A**) Experimental schematic for AstTau describing the work flow of 2D culture, oTau seeding, 3D assembloid, FKBP12 lentivirus transduction, and analysis at indicated data collection time points [week 1 (WK-1), WK-2, and WK-3]. (**B**) Representative images showing accumulation of phosphorylated tau (red, labeled by CP13, pTau Ser^202^) in oTau seeded asteroids and the reduction of pTau in FKBP12 overexpression conditions. The neurons were labeled by MAP-2 (violet), and astrocytes were labeled by glial fibrillary acidic protein (GFAP) (blue). Scale bars, 50 μm. (**C** and **D**) Quantification of FKBP12 and CP13-labeled fluorescence intensity normalized to the corresponding MAP-2 intensity as shown in (B). Data obtained from five independent asteroids. Error bars = SEM. Two-way ANOVA with Tukey’s multiple comparisons test was performed; *P < 0.01, ***P < 0.005, and ****P < 0.0001. (**E**) Representative images showing reduction in misfolding tau upon FKBP12 overexpression in brain assembloids after 3 weeks of oTau seeding. The misfolded tau was labeled with MC1 (red), neurons were labeled with Tuj1 (violet), and astrocytes were labeled with S100β (green) and DAPI used to label nuclei. Scale bars, 50 μm. (**F**) Quantification of MC1 labeled fluorescence intensity with normalization to corresponding levels of Tuj1. Data obtained from five independent asteroids. Error bars = SEM. Two-way ANOVA with Tukey’s multiple comparisons test was performed; *P < 0.01 and ****P < 0.0001. (**G**) Representative images showing toxic tau oligomers labeled with TOMA2 antibody (red) and degenerating neurons labeled with Fluoro-Jade B (green) in neuron-astrocyte assembloids with exposure to oTau and/or treated with FKBP12 overexpression for 3 weeks. Neurons were labeled with Tuj1 (violet) and FKBP12 labeled with antibody in bright blue. Scale bars, 50 μm. (**H** and **I**) Quantification of TOMA2 and Fluoro-Jade B labeled fluorescence intensity normalized to the corresponding Tuj1 intensity. Data obtained from five independent asteroids. Error bars = SEM. Two-way ANOVA with Tukey’s multiple comparisons test was performed; *P < 0.05, **P < 0.01, ***P < 0.005, and ****P < 0.0001.

The data presented in Fig. 7 indicate that FKBP12 changes the structure of tau fibrils, but the key question remaining is determining whether this change in tau fibril structure is good or bad for neurons. To address this key question, we used a 3D human neuron-astrocyte assembloid tauopathy model to investigate the effects of FKBP12 expression on tau-mediated neurodegeneration and examine effects on additional types of tau pathology (*45*). The 3D human neuron-astrocyte assembloid tauopathy model, termed AstTau, is a coculture system created by the Wolozin laboratory (*45*). AstTau is generated by propagating toxic human tau oligomers in human induced pluripotent stem cell (iPSC)–derived neurons in 2D culture and then combining with iPSC-derived astrocytes and growth in 3D culture to form human neuron/astrocyte assembloids (*45*). The AstTau system develops much of the neuronal and astrocytic pathology observed in tauopathies including misfolded, phosphorylated, oligomeric, and fibrillar tau; strong neurodegeneration; and reactive astrogliosis (*45*). The AstTau system allows testing whether FKBP12 protects against tau aggregation and neurodegeneration using a human-based culture system that models many aspects of tauopathies (*45*). We transduced the AstTau assembloids with FKBP12 lentivirus or a corresponding vector control starting from the first day of 3D culture followed by repeat lentiviral exposure every 4 days (when half the feeding medium was replaced). The design of the experiment is as shown in the scheme in Fig. 8A. The 3D brain assembloids were harvest at 1, 2, and 3 weeks after tau propagation, respectively, followed by the analysis of FKBP12 expression, tau pathology, and neurodegeneration (Fig. 8A). The results showed that FKBP12 lentivirus increased the expression level of FKBP12 by twofold at weeks 2 and 3 of the 3D culture (Fig. 8, B and C). The increase of FKBP12 robustly reduced hyperphosphorylation of tau labeled by the pTau Ser^202^ marker CP13 (Fig. 8, B to D). We also used the antibody MC1 to quantify levels of misfolded tau in AstTau system ± FKBP12 overexpression. Quantification of MC1 reactivity showed that FKBP12 strongly inhibited the accumulation of misfolded tau (Fig. 8, E and F). In parallel, we quantified neurodegeneration in AstTau, showing an equally strong decrease in Fluoro-Jade B labeling upon FKBP12 overexpression (Fig. 8, G to I). These result indicate that FKBP12 increased the neuronal resilience to extracellular stress and reduced neurodegeneration in the human AstTau model of tauopathy.

## DISCUSSION

To develop new therapeutic strategies for AD and other associated tauopathies, the identification and mechanistic understanding of proteins that can repair or remove abnormal tau deposits is essential. PPIases are a unique family of proteins that can markedly alter the conformation of tau both by their ability to catalyze cis-trans isomerization and by their chaperone activity. Regulations of these PPIases have been reported to affect the pathology of different neurodegenerative diseases, suggesting that an altered balance of these PPIases might contribute to the disease (*46*, *47*).

In this study, we showed that FKBP12 colocalizes with tau and its expression is reduced in the PS19 mice, in agreement with the reduction of FKBP12 in the AD brain. Our data further reveal that FKBP12 is translocated from axonal hillock to soma upon exposure to oTau that is again in synergy with the somatodendritic mislocalization of tau in AD. We also observed reduced accumulation of phosphorylated tau upon overexpression of FKBP12. FKBP12 acts as an anchoring protein of tau phosphatase calcineurin (*48*). Thus, together with calcineurin, FKBP12 might be able to dephosphorylate tau protein in the neurons.

As FKBP12 is a member of the PPIase family, the common notion is that it should mainly interact with the proline-rich region of tau. In contradiction to this notion, our data reveal that, although FKBP12 weakly senses a specific amino acid stretch in the proline-rich domain of tau, the main interaction site lies outside. Moreover, the interaction site of FKBP12 on tau is highly specific (^391^EIVYK^395^ and ^307^QIVYK^311^) unlike the case of other chaperones and PPIases. For example, the chaperone Hsp90 and the PPIase FKBP51 interact with a broad region comprising the P2 domain and the pseudo-repeats of tau (fig. S3) (*49*–*52*).

Our data also show that at an equimolar ratio of tau to FKBP12, the rate of tau aggregation slightly increases potentially due to the cis/trans isomerase activity of FKBP12. However, the presence of a higher concentration of FKBP12 delays the aggregation of tau due to its chaperone activity, leading to the formation of fibrils with different morphology. This kind of opposing effects at different concentrations was previously reported for the immunophilin CypA that increases the aggregation of α-synuclein via its PPIase activity and inhibits the aggregation via its chaperone activity (*53*). Although the effect of PPIase activity, as well as the chaperone activity of FKBP12 on the in vitro aggregation kinetics of tau, is not marked, it might explain the correlation behind the reduced expression of FKBP12 and the progression of the disease. In physiological conditions, the highly expressed FKBP12 can regulate the stability of monomeric tau through its chaperone activity via binding to ^306^VQIVYK^311^, i.e., one of the two specific FKBP12-binding motifs that overlaps with the aggregation-prone PHF6 motif. However, under pathological conditions, the hyperphosphorylated tau carrying phosphorylation at its tyrosine residues fails to bind to FKBP12 through its two specific structural motifs. The reduced levels of FKBP12 might then contribute to the formation of tau aggregates through its phosphorylation-independent PPIase activity.

In disease states, tau conformers and phosphorylation states are heterogeneous and dynamic. Because tau species that are not tyrosine phosphorylated are good substrates for FKBP12, increasing expression of FKBP12 can provide powerful protection against tau-mediated neurodegeneration. We demonstrate this principle using the AstTau system, which is a 3D neuron/astrocyte assembloid that recapitulates the major pathological states of tau seen in tauopathies, including tau misfolding, oligomerization, and fibrillization (*45*). Overexpressing FKBP12 strongly reduces tau pathology, which leads to a concomitant decrease in neurodegeneration. We note that FKBP12 acts on many proteins, which does raise the possibility that FKBP12 might provide additional neuroprotection through its actions on other pathways. The combined biophysical and physiological data complement each other to support the hypothesis that FKBP12 acts on tau to remove toxic conformations, protecting against tau-mediated neurodegeneration. Together, our study sheds light on the molecular mechanism behind the enigmatic role of FKBP12 in AD and other tauopathies.

## MATERIALS AND METHODS

### Animals

Use of all animals was approved by the Boston University Institutional and Animal Care and Use Committee (IACUC). All animals were housed in IACUC-approved vivariums at Boston University School of Medicine. PS19 P301S mouse breeders were purchased from the Jackson Laboratory and were breeding in-house and aged to aimed time point for experiment. Timed pregnant C57BL/6 were purchased from Charles River Laboratories and delivered at embryonic day 14 (E-14). In addition, the postnatal day 0 (P0) pups were used for primary hippocampal cultures.

### Extraction of S1p fractions containing oTau from P301S brain tissue

The frozen hippocampus and cortex tissues from 9-month-old PS19 P301S mice (100 to 250 mg) were weighed and put in a Beckman centrifuge tube with polycarbonate thick wall (catalog no. 362305). Then, 10× volume of homogenization buffer was used to homogenize brain tissue with Hsaio TBS (tris-buffered saline) buffer [50 mM tris (pH 8.0), 274 mM NaCl, and 5 mM KCl] supplemented with protease and phosphatase inhibitor cocktails (Roche, catalog nos. 05892791001 and 04906837001), as described previously (*21*). The homogenate was then ultracentrifuged at 28,000 rpm for 20 min at 4°C followed by aliquot the SN to a new Eppendorf tube labeled as S1 (TBS-soluble). Next, the SN (S1) fractions were ultracentrifuged a second time at 55,000 rpm at 4°C for 40 min. In addition, the TBS-extractable pellet (S1p) fractions were resuspended in 4× volume of TE buffer (10mM Tris, 1mM EDTA, pH 8.0) relative to the starting weight of the tissue homogenate.

The molecular weight of tau in the S1p fractions was documented by native PAGE, and the concentration of total tau was measured by Western blot using 3 to 12% reducing SDS-PAGE gel by comparison to a gradient concentrations of recombinant tau ladders, using the tau-5 antibody (detecting total tau) by immunoblot as described before (*21*). All the fractions were then normalized and divided into fractions of tau (20 μg/ml) for storage and future use.

### Primary cortical culture with P0 pups

Sterilized 12-mm glass coverslips were placed into each well of 24-well plate and then coated with 80 μl of poly-d-lysine (1 mg/ml; only on the coverslip) for 1 hour at room temperature (RT) in the culture hood. The plates were washed three times with sterile biology-grade water and dried in hood overnight covered in foil. For cortex dissection, P0 pups were anesthetized via hypothermia by wrapping in gauze and placing in aluminum foil pouch on ice. Then, the brain was isolated from the skull and separated into two hemispheres. The meninges from the brain tissue were completely removed before the cortex tissue was transferred to 0.25% trypsin-EDTA supplemented with 150 μl of deoxyribonuclease (DNase) for single-cell isolation. The tissue was resuspended in 2 ml of plating medium (minimum essential medium; Gibco, no. 11090; 2.5% fetal bovine serum, 1× penicillin/streptomycin, l-glutamine, and 0.6% d-glucose) and triturated gently with a 5-ml pipette. We then pass cells through a 70-μm cell strainer and count the cell number. We plated 60,000 cells per coverslip in 80 μl of medium (7.5 × 10^5^ cells/ml) for a 24-well plate. In addition, after 30 min of incubation, 1 ml of feeding medium (Neurobasal media, 1× B27 supplement, 1× penicillin/streptomycin, and 1× l-glutamine) was added into each well.

### Cell transduction

For cell transduction, AAV were added between days 2 and 5 to overexpress or knock down target protein. Briefly, at day 2, neurons were transduced with AAV1 vectors of human 4R0N wild-type tau at a multiplicity of infection (MOI) of 200. At day 5, neurons were transduced with AAV9 control or FKBP12 virus (MOI of 200). Approximately one-half volume of feeding medium was replaced every 3 to 4 days for cell maintenance until ready to use for experiment on days 14 to 21.

### Immunofluorescence labeling of fixed primary culture

Cells on coverslips in a 24-well were fixed with 0.5 ml of 4% paraformaldehyde/phosphate-buffered saline (PBS) for 15 min. The cells were washed three times in PBS, 5 min each wash. The cells were then permeabilized in 0.5 ml of PBS/0.1% Triton X-100 (PBST) for 15 to 30 min. Blocking was done in 0.5 ml of 5% bovine serum albumin (BSA) and 5% donkey serum in PBST for 1 hour. Primary antibody was incubated in diluted in 5% BSA/PBST overnight at 4°C and then washed three times in PBST, 10 min each. The cells were then incubated in secondary antibody diluted in 5% BSA/PBST, 2 hours at RT. All the secondary antibodies were purchased from Thermo Fisher Scientific made in donkey and used for 1:800 dilution in staining. After secondary antibody, cells were incubated in 4′,6-diamidino-2-phenylindole (DAPI) diluted 1:10,000 in PBST (stock solution of 5 mg/ml) for 5 min after first wash. Then, we washed the cells twice with PBST and once with PBS, 10 min each, before mounting coverslips. The coverslips with cells were then mounted on glass microscope slides using 8 to 10 μl of ProLong Gold Antifade mounting medium per coverslip. Slides were naturally dried in a fume hood (or store at 4°C until ready to dry in the fume hood). The primary antibodies used in this study for ICC are as follows: MAP-2 (chicken; AVES, MAP; 1:250), CP-13 (mouse; provided by P. Davies; 1:300), FKBP12 (rabbit; Thermo Fisher Scientific, catalog no. PA1-026A; 1:400). Anti–ankyrin-G antibody (N106/65), marking the axon initial segments/axon hillock, was purchased from Antibodies Incorporated, catalog no. 75-147.

### Immunofluorescence staining of fixed brain tissues

On the day of immune staining, selected sections with LEnt on bregma −2.8 were washed in PBS for 10 min and then permeabilized in 0.5 ml of PBS/0.25% Triton X-100 (PBST). The tissues was blocked in 5% BSA and 5% normal donkey serum in PBST, 1.5 to 2 hours at RT. Then, we diluted primary antibodies in 5% BSA/PBST and incubated sections for overnight at 4°C. On the second day, brain sections were washed three times in PBST, 15 min each followed by incubation in secondary antibodies (1:700 for DyLight/Alexa-conjugated antibodies made in donkey purchased from Thermo Fisher Scientific) in 5% BSA/PBST and incubated for 2 hours at RT. For DAPI nucleus stain, DAPI was diluted (1:10,000) in PBST followed by incubation for 15 min. The brain sections were mounted onto microscope glass slides in ProLong Gold antifade reagent. The primary antibodies used in this study for immunofluorescence labeling are as follows: CP-13 (mouse; provided by P. Davies, Northwell), 1:300; TOMA2 (Anti-Tau oligomer clone 2, mouse,provided by Rakez Kayed laboratory), 1:200; FKBP12 (rat; R&D Systems, catalog no. MAB3777), 1:500.

### Western blot

For the native page gel electrophoresis, mouse brain lysate were prepared with a native PAGE sample prep kit (catalog no. BN2008) and then run on Native PAGE Novex 3 to 12% bis-tris protein gels (Thermo Fisher Scientific, BN1003BOX) with a light blue cathode buffer. The molecular weight of tau aggregates was then detected by tau-5 antibody Western blot. For FKBP12 detection in primary culture, cell lysate was collected from frozen cultures with radioimmunoprecipitation assay lysis buffer. Reducing and nonreducing protein samples were separated by gel electrophoresis and transferred to 0.2-μm nitrocellulose membranes using a Bolt SDS-PAGE system (Life Technologies). Membranes were blocked in 5% nonfat dry milk in PBS supplemented with 0.025% Tween 20 (PBST) for 1 hour at RT, followed by incubation overnight at 4°C in primary antibody diluted in 5% BSA/PBST. Primary antibodies used were as follows: MAP-2 (rabbit; Millipore, AB5622; 1:5000); FKBP12 (rabbit; Thermo Fisher Scientific, catalog no. PA1-026A; 1:800). Membranes were then washed three times with PBST and incubated in horseradish peroxidase–conjugated secondary antibodies (Jackson ImmunoResearch) diluted in 1% BSA/PBST at RT for 1 hour. After incubation in secondary antibody, membranes were washed three times in PBST and developed using SuperSignal West Pico Chemilluminescent ECL substrate (Thermo Fisher Scientific, catalog no. 34080).

### 3D neuron-astrocyte brain assembloids

The detailed method, generation, and evaluation of the neuron-astrocyte assembloid tauopathy model, AstTau system, can be found in our recent publication (*45*). In brief, the following steps were performed.

#### NPC culture

Human iPSC-derived neural progenitor cells (NPCs; STEMCELL Technologies, catalog no. 70901) were maintained in serum-free STEMdiff Neural Progenitor Medium 2 (STEMCELL Technologies, catalog no. 08560) on the Corning Matrigel hESC-qualified Matrix (Corning, catalog no. 354277)–coated tissue culture plates. NPCs were plated at 50,000 cells/cm^2^ and passaged at 90% confluency by Accutase (STEMCELL Technologies, catalog no. 07920) dissociation as necessary. A full medium change was performed every other day. Low-passage (passage < 3) NPCs were cryopreserved in STEMdiff Neural Progenitor Medium 2 with 10% dimethyl sulfoxide (DMSO), and all NPCs used for experimentation were maintained at passage <6.

#### hiNC differentiation

NPCs were passaged and plated at 50,000 cells/cm^2^ in STEMdiff Forebrain Neuron Differentiation Media (STEMCELL Technologies, catalog no. 08600) on Corning Matrigel–coated tissue culture–treated plates and transduced with a NEUROG2 lentivirus (GeneCopoeia, catalog no. LPP-T7381-Lv105-A00-S) at an MOI of 3 to induce iPSC-derived neuronal cells (hiNCs). After 24 hours of transduction, a full medium change was performed.

#### hiAC differentiation

iPSC-derived astrocytic cells (hiACs) were differentiated from NPCs by small-molecule differentiation in STEMdiff Astrocyte Differentiation Media (STEMCELL Technologies, catalog no. cat100-0013) on Corning Matrigel–coated tissue culture–treated plates. A full medium change was performed daily for 4 days, and NPC cultures were passaged at 90% confluence by Accutase. NPCs were reseeded at 150,000 cells/cm^2^, and culture was continued in STEMdiff Astrocyte Differentiation Media with a full medium change every other day for 14 days, passaging as necessary with Accutase. At this stage, the differentiated astrocyte precursor cells (APCs) were cryopreserved in STEMdiff Astrocyte Differentiation Media with 10% DMSO. At time of experimentation, APCs were thawed and plated at 150,000 cells/cm^2^ in STEMdiff Astrocyte Maturation Media (STEMCELL Technologies, catalog no. 100-0016) on Corning Matrigel–coated tissue culture–treated plate. A full medium change was performed every other day for 6 days, with one passage by Accutase at 90% confluence as necessary.

#### Asteroid generation and maintenance

A single-cell suspension of hiNCs and hiACs was prepared by Accutase dissociation and washed once with Dulbecco’s modified Eagle’s medium (DMEM)/F-12 (STEMCELL Technologies, catalog no. 36254) to remove debris. hiNCs and hiACs were combined at a 1:1 ratio in Asteroid Media [DMEM/F-12 (STEMCELL Technologies, catalog no. 36254), 1% GlutaMAX (Thermo Fisher Scientific, catalog no. 35050061), 1% sodium pyruvate (Thermo Fisher Scientific, catalog no. 11360070), 1% N-2 supplement (Thermo Fisher Scientific, catalog no. 17502-048), 1% B-27 supplement (Thermo Fisher Scientific, catalog no. 17504044), 10 μM Y-27632 (EMD Millipore, catalog no. SCM075), 1% penicillin/streptomycin (Thermo Fisher Scientific, catalog no. 15140148), and heparin (1 mg/ml; Sigma-Aldrich, catalog no. H3149-250KU)] and plated in AggreWell800 microwells (STEMCELL Technologies, catalog no. 34815) coated with Anti-Adherence Rinsing Solution (STEMCELL Technologies, catalog no. 07010). The Aggrewell plate was immediately centrifuged at 100*g* for 3 min to capture the cells in the microwells and incubated for 24 hours. A half medium change was performed at 24 hours and then every other day for 1 week. At 1 week when the spheroids displayed, a smooth, bright edge under the cell culture microscope cultures was transferred to ultralow-attachment round-bottom 96-well plates (Thermo Fisher Scientific, catalog no. 07-201-680) and maintained in 100 to 200 μl of asteroid medium rotating at 85 rpm. A half medium change was performed every other day for up to 3 weeks. Cultures were monitored and live images captured using the AmScope or EVOS m7000 platforms.

#### hiNC oTau treatment

hiNCs were selectively exposed to oTau (0.04 mg/ml) by direct administration in cell culture medium for 24 hours before incorporation into asteroid culture.

#### FKBP12 lentivirus transduction

The mRFP-FKBP12 (Addgene, plasmid no. 67514; RRID:Addgene_67514) (*54*) and pLenti PGK FKBP12(F36V mutant)-OGT (Neo) (Addgene, plasmid no. 154294; RRID:Addgene_154294) (*55*) vector plasmids were obtained from Addgene. The two constructs were subcloned into the pHR lentiviral backbone with the spleen focus-forming virus (SFFV) promoter, respectively, to generate the FKBP12 and mutant FKBP12 (F36V) plasmids. The In-Fusion Cloning Kit (Takara) was used for the subcloning, and the resulting constructs were fully sequenced to confirm the absence of unwanted substitutions. The lentivirus was prepared as described previously (*56*): In brief, the human embryonic kidney–293T cells were plated at a concentration of 1 × 10^6^ cells per well in a six-well plate. Eighteen hours later, cells were transiently cotransfected with PSP (1200 ng), VSV-G (400 ng), and target (400 ng) plasmids using 6 μl of FuGene HD (Promega). Seventy-two hours later, conditioned medium was harvested and centrifuged at 1000*g* for 5 min to remove dead cells and debris. SN was stored at −80°C until use. For primary neuron transduction, lentivirus was concentrated 10× using a lenti-X concentrator (Clontech) with the concentrated pellet being resuspended in PBS with 25 mM Hepes (pH 7.4). The concentrated lentivirus was added into the feeding medium for brain assembloids at the dilution of 20 μl/ml.

#### Immunofluorescence labeling

Selected brain assembloids from each condition were washed in PBS in a U-bottom 96-well plate and permeabilized by PBS/0.01% Triton X-100 (PBST). The tissue was then blocked in PBST supplemented with 5% BSA and 5% normal donkey serum for 1.5 to 2 hours at RT. After blocking, primary antibodies diluted in 5% BSA/PBST were incubated with tissue for overnight at 4°C. On the second day, the AstTau were washed three times in PBST, 15 min each, before they were transferred into secondary antibodies dilute (1:700 of DyLight/Alexa-conjugated antibodies made in donkey purchased from Thermo Fisher Scientific in 5% BSA/PBST) for 2 hours at RT. For DAPI nucleus stain, DAPI (1:10,000) was diluted in PBST and incubated with asteroids for 15 min followed by washes with PBST (two times) and then with PBS (one time), 10 min each. The assembloids were then mounted onto microscope glass slides in ProLong Gold antifade reagent (Thermo Fisher Scientific, catalog no. P36930) and stored in the dark until imaging. Primary antibodies used for brain assembloids labeling were as follows: Tuj1/βIII-Tubulin (chicken; Synaptic Systems, catalog no. 302 306’ 1:300), MAP-2 (rabbit; Millipore, catalog no. AB5622; 1:1000), rabbit monoclonal anti-S100β (Abcam, catalog no. ab52642; 1:400); glial fibrillary acidic protein monoclonal antibody (Thermo Fisher Scientific, catalog no. 13-0300, 1:400), mouse monoclonal anti-TOMA2 (provided by R. Kayed; 1:300), MC1 (provided by P. Davies, Northwell; 1:300), CP-13 (provided by P. Davies, Northwell; 1:300), and FKBP12 (Thermo Fisher Scientific, catalog no. PA1-026A; 1:300).

#### Fluoro-Jade B staining

The Fluoro-Jade B reagent was purchased from EMD Millipore (catalog no. AG310-30MG), and the staining protocol was followed as instructed by the manufacturer. Briefly, the staining solution was prepared from a 0.01% stock solution for Fluoro-Jade B that was made by adding 10 mg of the dye powder to 100 ml of distilled water. To make up 100 ml of staining solution, 4 ml of the stock solution was added to 96 ml of 0.1% acetic acid vehicle. This results in a final dye concentration of 0.0004%. The stock solution, when stored in the refrigerator, was stable for months, whereas the staining solution was typically prepared within 10 min of use and was not reused. Before staining, the asteroids were rinsed in distilled water and were then treated with 0.06% KMnO_4_ solution for 15 min. Then, the asteroids were stained with Fluoro-Jade B working solution for 30 min followed by being washed with PBS twice for 5 min each. Brain assembloids were mounted in ProLong Gold antifade reagent (Thermo Fisher Scientific, catalog no. P36930) and stored in the dark until imaging.

### Image analysis

Images were captured by Carl Zeiss confocal LSM 700 and confocal Zeiss LSM 880 with Airyscan. The high-magnification images of primary neurons were captured by confocal Zeiss LSM 880 with Airyscan mode. The immunofluorescence-labeled neurons in each image from primary cultures were quantified by ImageJ with function of automatically cell counting. The dendritic length measurement of neurons in MAP-2 staining was using ImageJ plug-ins NeuronJ to trace the MAP-2–positive processes. Colocalization of TOMA2-positive tau oligomers to FKBP12 granules in neuronal soma was analyzed by Fiji (ImageJ) coloc2 plug-in.

### Protein preparation

Unlabeled, ^15^N-labeled, and ^13^C-^15^N–labeled 2N4R tau (hTau40, UniProt ID 10636-8, 441 residues) or ^15^N-labeled Y394N 2N4R tau was expressed in *Escherichia coli* strain BL21(DE3) from a pNG2 vector (a derivative of pET-3a, Merck-Novagen, Darmstadt) in the presence of ampicilin. In the case of unlabeled protein, cells were grown in 6 liters of LB and induced with 0.5 mM isopropyl-β-d-thiogalactopyranoside (IPTG) at an optical density at 600 nm (OD_600_) of 0.8 to 0.9. To obtain ^15^N-labeled protein or ^13^C-^15^N–labeled protein, cells were grown in LB until an OD_600_ of 0.6 to 0.8 was reached, then centrifuged at low speed, washed with M9 salts (Na_2_HPO_4_, KH_2_PO_4_, and NaCl) and resuspended in minimal medium M9 supplemented with ^15^NH_4_Cl (1 g/liter) as the only nitrogen source and ^13^C-glucose (4 g/liter) as the carbon source, and induced with 0.5 mM IPTG. Subsequently, the bacterial cells were harvested by centrifugation, and the cell pellets were resuspended in lysis buffer [20 mM MES (pH 6.8), 1 mM EGTA, 2 mM dithiothreitol (DTT)] complemented with protease inhibitor mixture, 0.2 mM MgCl_2_, lysozyme, and DNAse I. Next, cells were disrupted with a French pressure cell press (in ice-cold conditions to avoid protein degradation), NaCl was added to a final concentration of 500 mM, and lysates were boiled for 20 min. Denatured proteins were removed by ultracentrifugation with 127,000*g* at 4°C for 30 min. To precipitate the DNA, streptomycin sulfate (20 mg/ml) was added to the SN and incubated for 15 min at 4°C followed by centrifugation at 15,000*g* for 30 min. The pellet was discarded, and tau protein was precipitated by adding ammonium sulfate (0.361 g/ml) to the SN, followed by centrifugation at 15,000*g* for 30 min. The pellet containing tau protein was resuspended in buffer A [20 mM MES (pH 6.8), 1 mM EDTA, 2 mM DTT, 0.1 mM phenylmethylsulfonyl fluoride (PMSF), and 50 mM NaCl] and dialyzed against the same buffer (buffer A) to remove excess salt. The following day, the sample was filtered and applied onto an equilibrated ion exchange chromatography column, and the weakly bound proteins were washed out with buffer A. The protein was eluted with a linear gradient of 60% final concentration of buffer B [20 mM MES (pH 6.8), 1 M NaCl, 1 mM EDTA, 2 mM DTT, and 0.1 mM PMSF]. Unlabeled protein samples were concentrated by ultrafiltration (5 kDa; Vivaspin, Sartorius) and purified by reverse-phase chromatography using a preparative C4 column (Vydac 214 TP, 5 μm, 8 mm by 250 mm). The purified unlabeled protein was lyophilized and redissolved in the buffer containing 25 mM Hepes, 10 mM KCl, 5 mM MgCl_2_, and 0.01% NaN_3_ (pH 7.2). In case of ^15^N-labeled or ^13^C-^15^N–labeled protein, the protein was collected after Mono S, concentrated by ultracentrifugation (5 kDa; Vivaspin, Sartorius), and further purified by gel filtration chromatography using an SD 75 26/600 column (GE Healthcare).

Human FKBP12 was cloned into a pET28a plasmid with a Tobacco Etch Virus (TEV) protease cleavable N-terminal His tag and transformed into BL21 competent cells. Inoculated LB cultures were grown to an OD_600_ of 0.8 and induced with 1 mM IPTG, as previously described (*52*). The sample was spun down at 3500*g* for 30 min, and the pellet was resuspended in nickel chromatography running buffer [20 mM tris-HCl (pH 8.0), 0.5 mM NaCl, and 10 mM imidazole] and stored at −80°C. Pellets were then thawed and sonicated to lyse cells. Samples were centrifuged at 50,000*g* for 1 hour to isolate SN, and a standard gravity nickel column was performed. TEV protease (2 mg/ml, 1 ml) was then added to the elution fraction for 4 to 7 hours at RT. The solution was then dialyzed back into nickel chromatography running buffer followed by a quick spin for 5 min at 4000*g* to remove any precipitation before running a second nickel column. Size exclusion chromatography was then performed using a HiLoad 16/600 Superdex 200 pg column, and fractions were pooled, concentrated, flash frozen, and stored at −80°C.

### Protein phosphorylation

To phosphorylate tau, 200 μM ^15^N-labeled 2N4R tau was incubated at 30°C for 12 hours in an Eppendorf thermomixer with 300-rpm shaking in the presence of 1 μM cAbl enzyme (PR4348B, Thermo Fisher Scientific) and 5 mM adenosine 5′-triphosphate, 2 mM EGTA, 1 mM PMSF, and 5 mM MgCl_2_. After 12 hours of reaction, the sample was boiled at 98°C for 20 min to precipitate the enzyme, followed by centrifugation at 20,000*g* in an Eppendorf centrifuge 5424. Next, the pellet was discarded, and the SN containing phosphorylated tau was dialyzed against the buffer containing 50 mM NaP, 10 mM NaCl, and 1 mM TCEP (tris(2-carboxyethyl)phosphine) (pH 6.8).

### Aggregation assays

Aggregation assays of 2N4R tau without or with FKBP12 were performed using the previously described co-factor-free aggregation protocol (*37*). Briefly, 25 μM protein, either in the absence or presence of equimolar or two/fivefold excess of FKBP12, was aggregated at 37°C in the buffer containing 25 mM Hepes, 10 mM KCl, 5 mM MgCl_2_, 3 mM TCEP, 0.01% NaN_3_ (pH 7.2) (aggregation assay buffer) in a 96-well plate using a Tecan spark plate reader. Three polytetrafluoroethylene(PTFE) beads along with double orbital shaking were used to promote the aggregation. ThT at a final concentration of 50 μM was used to monitor the aggregation kinetics.

To perform in vitro seeding experiments, cofactor-free 2N4R tau fibrils were used as seeds. Seeds (2%; w/w) were added to 25 μM monomeric 2N4R tau protein either in the absence or presence of equimolar, two- or fivefold molar excess of FKBP12 in the aggregation assay buffer. Before addition, the seeds were sonicated for 2 min at 37°C in a water bath. The same protocol was used for aggregation, as described above. All measurements were performed with three independently prepared samples.

### Circular dichroism

Fifty microliters of 25 μM 2N4R fibril either in the absence or presence of fivefold excess of FKBP12 was centrifuged at 20,000*g* for 15 min in an Eppendorf centrifuge 5424. The SN was removed, and the pellet was resuspended in 50 μl of distilled water. The fibrils were transferred to a 0.02-cm pathlength cuvette, and CD data were collected from 190 to 260 nm using a Chirascan-plus qCD spectrometer (Applied Photophysics, UK) at 25°C, 1.5 s per point in 1-nm steps. The datasets were averaged from 10 repeated measurements. Spectra were baseline-corrected and smoothed with a window size of 4.

### Electron microscopy

Forty microliters of 25 μM 2N4R tau fibrils either in the absence or presence of fivefold excess of FKBP12 was pelleted down by centrifugation at 20,000*g* using an Eppendorf centrifuge 5424. The SN was discarded, and the pellet was redissolved in 30 μl of the buffer containing 25 mM Hepes, 150 mM KCl, 5 mM MgCl_2_, and 0.01% NaN_3_ (pH 7.2). Next, the fibrils were sonicated in a water bath (Bandelin sonorex) for 3 min. After sonication, 5.5 μl of fibril sample was mixed with 0.5 μl of pronase protease (1 mg/ml; 53702, Merck Millipore) followed by adsorbing onto carbon-coated copper grids. The samples were stained with 1% uranyl acetate solution, and the images were taken with a Tietz F416 CMOS camera (TVIPS, Gauting, Germany) using a CM 120 transmission electron microscope (FEI, Eindhoven, The Netherlands).

### NMR spectroscopy

^1^H-^15^N HSQC spectra of uniformly ^15^N-labeled 2N4R tau (45 μM) in the absence or presence of unlabeled FKBP12 (90 or 225 μM) were recorded in the buffer containing 50 mM NaP, 10 mM NaCl, and 1 mM DTT (pH 6.8) at 278 K on an Avance III 600 MHz spectrometer (Bruker) using a 5 mm QCI (H/C/N/F) cryoprobe. The spectra were collected with 64 scans per point (nanoseconds) and indirect acquisition times td1 = 17.5 ms and td2 = 15.5 ms.

^1^H-^15^N HSQC spectra of uniformly ^15^N-labeled phosphorylated 2N4R tau (30 μM) or Y394N 2N4R tau (30 μM) without or with unlabeled FKBP12 (60 or 150 μM) were recorded in the buffer containing 50 mM NaP, 10 mM NaCl, and 1 mM DTT (pH 6.8) at 278 K on an Avance neo 800 MHz spectrometer (Bruker) using a 3 mM QCI (H/C/N/F) cryoprobe. The spectra were collected with 40 scans per point (nanoseconds) and indirect acquisition times td1 = 12 ms and td2 = 9 ms.

The ^1^H-^13^C HSQC spectrum of uniformly ^13^C-^15^N–labeled 2N4R tau (100 μM) was recorded in the buffer 50 mM NaP, 10 mM NaCl, and 1 mM DTT (pH 6.8) at 278 K on an Avance neo 800 MHz spectrometer (Bruker) using a 3 mM QCI (H/C/N/F) cryoprobe. The spectrum were collected with 64 scans and indirect acquisition times td1 = 16 ms and td2 = 9.8 ms.

The chemical shift assignments of 2N4R tau had been previously established (*26*). The spectra were recorded using Topsin 3.6.2 software (Bruker) and analyzed with CCPNMR 2.4.2 software (*57*).

Residue-specific intensity ratios were calculated according to intensity ratio = *I*/*I*_0_, where *I* is the intensity of cross peaks in the 2D ^1^H-^15^N HSQC spectrum of 2N4R tau in the presence of twofold (or fivefold) excess of FKBP12, and *I*_0_ is the intensity of the cross peaks of 2N4R tau alone. The chemical shift perturbation (CSP) was calculated according to

$$\mathrm{CSP}=\sqrt{0.5[{\left(\Delta H\right)}^{2}+{\left(\Delta N/5\right)}^{2}]}$$

### Computational analysis

To gain insight into the structure of the complex of FKBP12 with the tau sequences, which NMR showed to bind to FKBP12, we modeled the complex using Alphafold2 (*22*). To do so, we connected the FKBP12 sequence (UniProt: P62942) to either the 11-residue fragment ^287^DHGAEIVYKSP^397^ or the 11-residue fragment ^303^GGSVQIVYKPV^313^ using a 300-residue poly-glycine linker (*27*). Each merged sequence was submitted to the Alphafold2 server at https://colab.research.google.com/github/sokrypton/ColabFold/blob/main/AlphaFold2.ipynb, generating five model structures of the two complexes each. The structures were analyzed using the open-source molecular visualization software PyMOL (Schrödinger).

The impact of mutations on the stability of the complex of tau’s ^307^QIVYK^311^ and ^391^EIVYK^395^ with FKBP12 was predicted using DynaMut2 (*32*). Previous analysis showed that AlphaFold2 structures that are predicted with high confidence values (as is the case for the ^07^QIVYK^311^/FKBP12 and ^391^EIVYK^395^/FKBP12 complex structures) are as useful for estimation of mutation-associated stability changes as experimentally determined protein structures (*58*).

### Statistical analysis

Statistical analyses were performed using GraphPad Prism version 6.00 for Windows with two sided α of 0.05. All group data are expressed as means ± SEM. Colum means were compared using one-way analysis of variance (ANOVA) with treatment as the independent variable. In addition, group means were compared using two-way ANOVA with factors on genotype and fractions treatment, respectively. When ANOVA showed a significant difference, pair-wise comparisons between group means were examined by Tukey’s, Dunnett, or uncorrected Fisher’s least significant difference multiple comparisons test.

## Notes

View/request a protocol for this paper from *Bio-protocol*.
