# Supplementary material for: Chaperoning of specific tau structure by immunophilin FKBP12 regulates the neuronal resilience to extracellular stress
Source: Sci Adv. 2023 Feb 1;9(5):eadd9789. doi: 10.1126/sciadv.add9789 (PMC9891691; doi:10.1126/sciadv.add9789)
Supplement: Supplementary file 1 — Figs. S1 to S3 [file sciadv.add9789_sm.pdf]

Science Advances
AAAS

Supplementary Materials for

Chaperoning of specific tau structure by immunophilin FKBP12 regulates the neuronal resilience to extracellular stress

Lulu Jiang *et al.*

Corresponding author: Benjamin Wolozin, bwolozin@bu.edu; Markus Zweckstetter, Markus.Zweckstetter@dzne.de

Sci. Adv. **9**, eadd9789 (2023)
DOI: 10.1126/sciadv.add9789

This PDF file includes:

Figs. S1 to S3

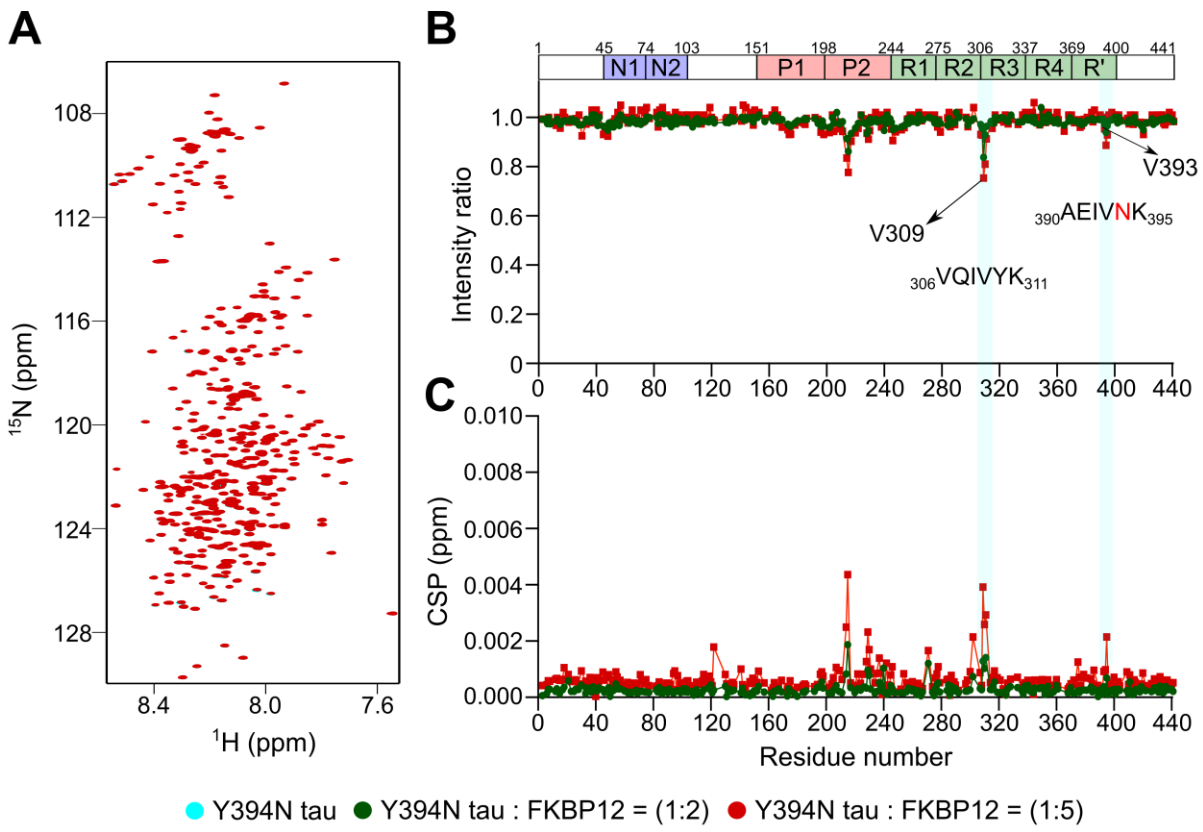

Supplementary Figure 1. Interaction between Y394N tau and FKBP12. A, 2D ^{1}H-^{15}N HSQC spectra of Y394N tau in the absence (cyan) or five-fold excess (red) of FKBP12. **B,C** Changes in the intensities (B) and chemical shift perturbations (C) of cross-peaks in the HSQC spectra of Y394N tau upon addition of two-fold (green) and five-fold (red) excess of FKBP12. The domain organization of tau is shown on top.

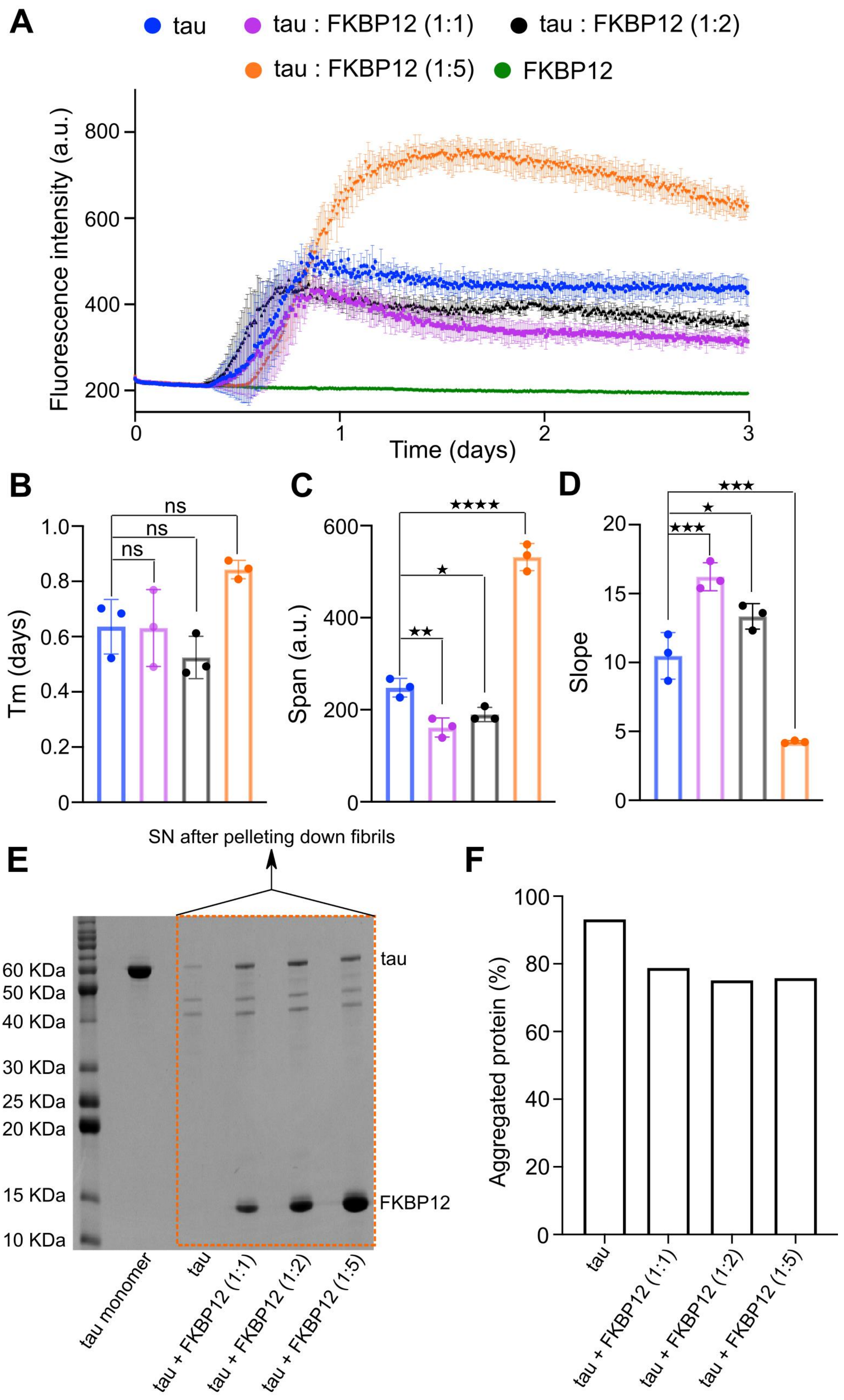

A
tau
tau : FKBP12 (1:1)
tau : FKBP12 (1:2)
tau : FKBP12 (1:5)
FKBP12
Fluorescence intensity (a.u.)
Time (days)
B
Tm (days)
ns
C
Span (a.u.)
D
Slope
E
SN after pelleting down fibrils
60 KDa
50 KDa
40 KDa
30 KDa
25 KDa
20 KDa
15 KDa
10 KDa
tau
FKBP12
tau monomer
tau + FKBP12 (1:1)
tau + FKBP12 (1:2)
tau + FKBP12 (1:5)
F
Aggregated protein (%)

Supplementary Figure 2. Effect of FKBP12 on the *de novo* aggregation of tau. A. Aggregation kinetics of 25 µM tau in the absence (blue) or presence of equimolar (purple), 2-fold (black) or 5-fold (orange) excess of FKBP12. 125 uM of FKBP12 (5-fold molar excess of tau) didn't aggregate (green) even after incubation for three days. Error bars represent standard deviation of three independent samples. **B**. Half time of aggregation (Tm) of tau in the absence (blue) or presence of equimolar (purple), 2-fold (black) or 5-fold (orange) excess of FKBP12. Data are represented as mean ± standard deviation of n=3 samples. The statistical analysis was performed by one-way ANOVA. **C**. Span of Thioflavin-T intensity in the aggregation curves of tau in the absence (blue) or presence of equimolar (purple), two-fold (black) or five-fold (orange) FKBP12 excess. Data are represented as mean ± standard deviation of n=3 samples. The statistical analysis was performed by one-way ANOVA. *p = 0.0305, **p = 0.0036, ****p<0.0001 **D**. Rate of elongation of tau fibrils (slope) without (blue) or with equimolar (purple), two-fold (black) or five-fold (orange) excess of FKBP12. Data are represented as mean ± standard deviation of n=3 samples. The statistical analysis was performed by one-way ANOVA. * p = 0.0305, ***p [tau vs tau+FKBP12 (1:1)] = 0.0005, ***p [tau vs tau+FKBP12 (1:5)] = 0.0003 **E,** SDS-PAGE gel of the monomeric tau protein and the supernatant (SN) of the tau fibrils (after centrifugation) either in the absence or presence of equimolar, two-fold, or five-fold excess of FKBP12. The fibril samples were taken after the three days of aggregation, shown in (A). **F,** Quantification of aggregated tau. The % of aggregated tau protein was determined by dividing the intensity of the supernatant (SN) to the monomeric tau.

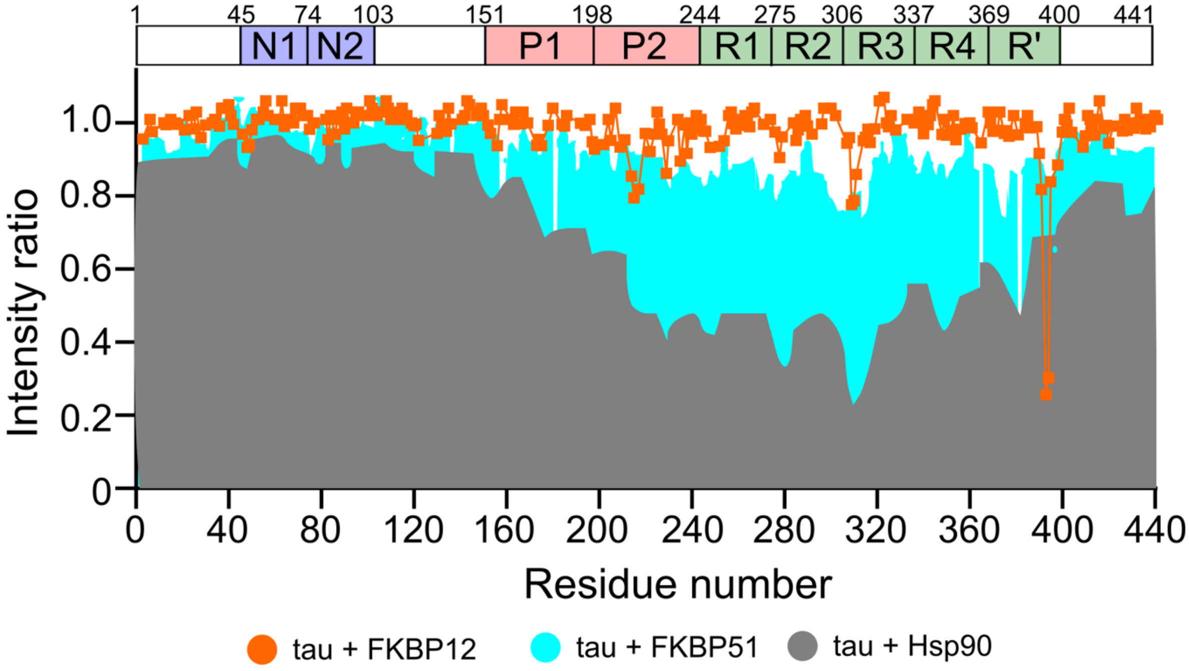

Supplementary Figure 3. Comparison between the interaction of FKBP12, FKBP51, and Hsp90 with tau. Intensity ratio broadening of tau in the presence of FKBP12 (orange), FKBP51 (cyan), and Hsp90 (grey). The intensity ratio plot of tau in the presence of FKBP51 & Hsp90 is taken from Baker et al (**Reference #52 in main manuscript**) & Karagöz et al (**Reference #49 in main manuscript**), respectively.
